# Structural Exploitation of Cinnarizine Identified Novel Drug-Like Anthelmintic Agents Against *Angiostrongylus cantonensis*

**DOI:** 10.1021/acsinfecdis.5c00634

**Published:** 2025-10-01

**Authors:** Bruna L. Lemes, Mariana A. Siegl-Breno, Mikaelly K. Silva-Nunes, Flavia B. Lopes, Aline S. Silva, Natalia E. P. Motta, Josué de Moraes, João Paulo S. Fernandes

**Affiliations:** † Research Center on Neglected Diseases, 92928 Guarulhos University, Guarulhos 07023-070, Brazil; ‡ Department of Pharmaceutical Sciences, 28105 Federal University of São Paulo, Diadema 09913-030, Brazil; § Research Center on Neglected Diseases, Scientific and Technological Institute, 149945 Brazil University, São Paulo 08230-030, Brazil; ∥ Department of Medicine, Federal University of São Paulo, São Paulo 04023-062, Brazil

**Keywords:** anthelmintic agents, molecular simplification, drug-likeness, angiostrongyliasis

## Abstract

The impact of helminthiases on global health for both humans and animals and the limited efficacy of existing drugs against these infections reinforces the urgent need for novel anthelmintic agents. On this background, in previous work we identified cinnarizine, a first-generation antihistamine, as effective anthelmintic agent against *Angiostrongylus cantonensis* first-stage larvae (L1) in vitro. *A. cantonensis* worm is the causative agent of neuroangiostrongyliasis, a condition that leads to eosinophilic meningitis with no effective treatment to date. In the present work, modifications on cinnarizine structure were designed to improve its efficacy against the larvae but keeping its ability to cross the blood brain barrier allied to improvement in the drug-like and solubility profile. A set of 11 compounds were synthesized and tested against L1 larvae, showing EC_50_ values ranging from 9.3 to 4.2 μM. The most effective were also tested against infective third-stage larvae (L3), with EC_50_ 18.1–8.6 μM. None of the compounds showed toxicity to both HaCat mammalian cells (at 500 μM) and *Caenorhabditis elegans* (at 1000 μM), indicating their high selective toxicity toward *A. cantonensis*. Structure–activity relationship analysis using molecular descriptors indicated that presence of two basic nitrogen atoms and balanced lipophilicity of compound **2b** (EC_50_ L1 9.3 μM; L3 8.8 μM) played the role in the anthelmintic activity, and simplified compound **3a** (EC_50_ L1 8.7 μM; L3 18.1 μM) represent a novel prototype for further modifications.

## Graphical abstract

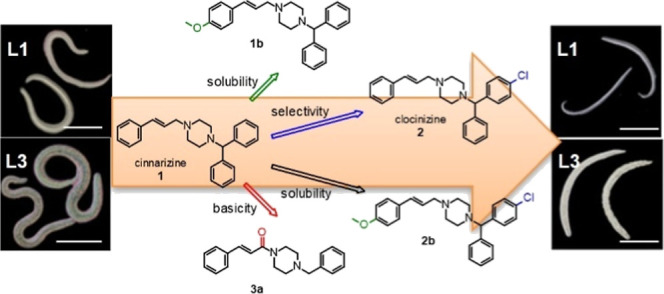

Helminthiases are parasitic worm infections that have a significant global health impact due to their high prevalence, especially among poor and marginalized populations. It is estimated that around 1.5 billion people are infected with at least one species of parasitic helminth, many of which are of zoonotic origin.^1,2^ Despite this burden, few new anthelmintic drugs have entered clinical use in recent decades, and current treatment options remain limited,^3^ with notable exceptions being the FDA approvals of moxidectin for onchocerciasis and triclabendazole for fascioliasis.^4,5^ Recognizing this challenge, the World Health Organization (WHO) launched a roadmap to control or eliminate several helminthiases by 2030.^6^ The discovery of new anthelmintic agents is one of the core strategies outlined in this plan.

Among emerging helminth infections, the nematode *Angiostrongylus cantonensis* stands out due to its ability to cause serious neurological disease in humans. Known as the rat lungworm, *A. cantonensis* is a parasitic nematode whose natural definitive hosts are rats, in which the adult worms reside in the pulmonary arteries and right ventricle.^7^ The female worms lay eggs that hatch into first-stage larvae (L1) within the pulmonary vasculature; these larvae migrate to the pharynx, are swallowed, and are ultimately excreted in the feces. Terrestrial gastropods such as snails and slugs serve as intermediate hosts, becoming infected upon ingesting L1 larvae. Humans become accidental hosts by ingesting L3 larvae through consumption of raw or undercooked infected gastropods—paratenic hosts (e.g., freshwater shrimp, crabs)—or contaminated produce.^8^ Once in the human host, the larvae migrate to the central nervous system (CNS), causing eosinophilic meningitis as clinical outcome (neuroangiostrongyliasis).^9^ Despite its rising public health significance, neuroangiostrongyliasis has still no specific treatment, which is done through palliative administration of corticosteroids since albendazole and other anthelmintics have limited efficacy against *A. cantonensis*.^7,10^

In a previous study, we screened a set of clinically available antihistamines against *A. cantonensis* L1 larvae, resulting in promising prototypes for further investigation, including cinnarizine.^11^ Cinnarizine is a first-generation antihistamine that permeates the blood brain barrier (BBB),^12^ making it a candidate for treating CNS infections such as neuroangiostrongyliasis. However, its effects on CNS may limit its therapeutic utility, highlighting the need for structural modifications aimed at improving its pharmacokinetic profile while preserving anthelmintic activity.

The structural simplicity of cinnarizine (**1**) motivated us to explore the structural features that could play the role on its anthelmintic activity, considering the hypothesis depicted in Figure 1. These modifications were planned to keep the anthelmintic activity allied to an improvement in the drug-like profile, as well as modulating its potential side effects on CNS. For that, in this work a set of 11 analogues of cinnarizine were designed and a preliminary in silico analysis was carried out to evaluate their drug-likeness. The compounds were prepared, evaluated for their in vitro activity against *A. cantonensis* larvae and initial structure–activity relationships were determined using statistical methods.

**Figure 1:**
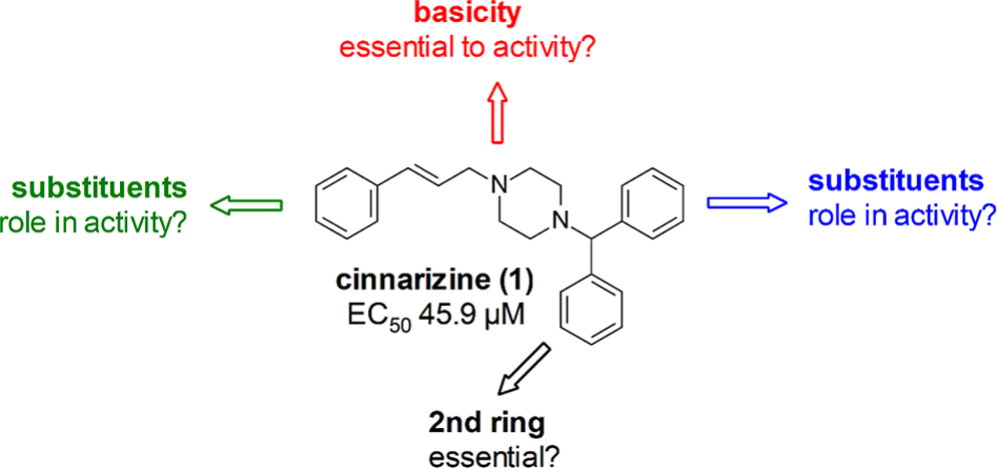
Hypothesis designed from cinnarizine’s structure to explore the anthelmintic activity.

## Results and Discussion

The current management of neuroangiostrongyliasis lacks a specific, evidence-based drug and care is primarily supportive and done by administration of corticosteroids to mitigate the inflammatory response caused during larval death.^7,10^ This therapy does not shorten the course of infection, and the administration of an anthelmintic agent is worth of consideration, but still debated. Currently, cotherapy of corticosteroids with albendazole has been employed in some cases of neuroangiostrongyliasis (including for prophylaxis in endemic areas),^8,13^ but the benefits of the anthelmintic are inconsistent. Considering the limited efficacy of albendazole against *A. cantonensis* and its pharmacokinetic contrains, the search for novel drugs with this potential is still needed.

The identification of anthelmintic effects caused by some antihistamines raises interesting possibilities, mainly because these drugs could block the histamine-related inflammatory effects in the human host during larval death and provide synergistic effect associated with antiparasitic activity. Fox et al.^14^ argued that the blockade of histamine H_1_ receptor could improve the treatment of endoparasitic infections in a murine filariasis model, but to date there is no support to the clinical use of antihistamines during parasitic infections. In this context, our prior findings of anthelmintic activity derived from antihistamines against *A. cantonensis* ^11^ and *Schistosoma mansoni* ^15^ motivated a structural exploitation of cinnarizine by designing analogues with antiparasitic effects but with different mode of action from the clinically available anthelmintics. The structural modifications can also help to unveil whether the antihistaminic activity is related to the effects against the larvae in vitro.

The compounds (Figure 2) were designed using the hypothesis presented in Figure 1. Starting from cinnarizine’s structure, we first considered simplifying the structure by removing one of the rings on the benzhydryl moiety, generating the analogue **1a**. This modification improved the predicted solubility (Table 1) and allowed evaluation of the role played by the second aromatic ring in the anthelmintic activity. Additionally, to explore how modifications to the cinnamyl moiety could affect activity, we introduced a methoxy group (analogue **1b**), aiming to maintain the lipophilicity of cinnarizine while altering the electron density of the aromatic ring.

**Figure 2:**
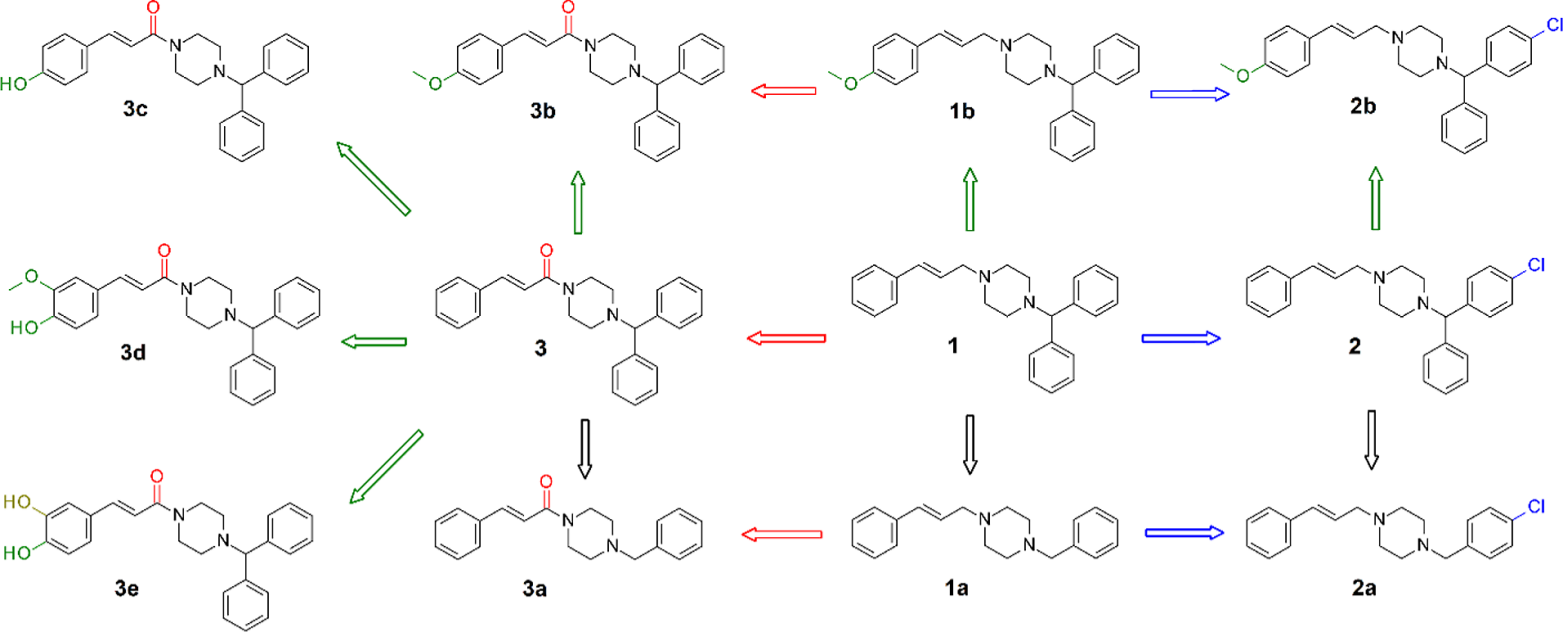
Compounds designed starting from cinnarizine through simplification (black arrows), modifications in the cinnamyl (green arrows) and benzhydryl (blue arrows) rings and elimination of basicity (red arrows) of the piperazine nitrogen.

**Table 1:** In Silico Estimation of logP, Solubility, and Drug-likeness Descriptors for the Designed Compounds

compound	MW	Fsp3	RB	TPSA	MR	logP^a^	logS^b^	GIT absorption^c^	BBB permeant^c^
**1**	368.51	0.23	6	6.48	125.86	4.72	–5.84	high	yes
**1a**	292.42	0.30	5	6.48	101.37	3.53	–4.29	high	yes
**1b**	398.54	0.26	7	15.71	132.35	4.70	–5.91	high	yes
**2**	402.96	0.23	6	6.48	130.87	5.25	–6.43	high	yes
**2a**	326.86	0.30	5	6.48	106.38	3.98	–4.65	high	yes
**2b**	432.98	0.26	7	15.71	137.36	5.20	–6.39	high	yes
**3**	382.50	0.19	6	23.55	126.06	4.18	–5.31	high	yes
**3a**	306.40	0.25	5	23.55	101.57	2.94	–3.76	high	yes
**3b**	412.52	0.22	7	32.78	132.55	4.15	–5.38	high	yes
**3c**	398.50	0.19	6	43.78	128.08	3.73	–5.16	high	yes
**3d**	428.52	0.22	7	53.01	134.57	3.78	–5.25	high	yes
**3e**	414.50	0.19	6	64.01	130.10	3.38	–5.03	high	yes

a Average of five different methods implemented in SwissADME.

b ESOL topological method; logS > −4.0 = soluble; −4.0 > logS > −6.0 = moderately soluble; logS < −6.0 = poorly soluble.

c Estimated by the BOILED-Egg model.

To assess whether affinity for the histamine receptor is correlated to the observed activity, we inserted a chlorine atom into the benzhydryl moiety—an alteration known to enhance antihistaminic activity in cinnarizine analogues.^11,16^ Clocinizine (**2**) a chlorinated derivative of cinnarizine, previously showed increased efficacy against *S. mansoni* ^15^ and was therefore included in our set, along with its simplified (**2a**) and methoxylated (**2b**) analogues.

Finally, the role of nitrogen basicity, important to the antihistamine activity of cinnarizine, was assessed by designing amide analogues (**3a–3e**) with the similar modifications (**3a–3b**) and additional analogues with polar substituents in the cinnamoyl moiety (**3c–3e**) were designed to increase solubility allied to modification in the electron density of the ring.

In silico analyses were performed using the SwissADME online platform^17^ to estimate logP, solubility and drug-likeness descriptors of the compounds. As shown in Table 1, all designed compounds display equilibrated lipophilicity (determined by the TPSA/WlogP relationship), suggesting adequate properties for BBB penetration. The simplified compounds **1a**, **2a** and **3a** also showed a clear improvement in solubility and drug-likeness compared to their benzhydryl-containing counterparts, while analogues **3c–3e** present improved solubility than **3b**.

The compounds were synthesized as shown in Scheme 1. Amines **1a**, **2** and the protected precursor **5a** were prepared by alkylation of substituted piperazines (**4a–d**) with cinnamyl chloride in good yields.^15^ Compound **2a** was prepared from Boc-deprotected compound **5b** using 4-chlorobenzyl chloride using a similar method. Analogues **1b** and **2b** were prepared by reductive amination of 4-methoxy-cinnamaldehyde with substituted benzhydryl piperazines (**4a–b**) as previously described.^15^ The benzhydryl piperazine **4a** was obtained by reacting with an excess of anhydrous piperazine with benzhydryl chloride obtained in situ from a one-pot method, using benzhydryl alcohol and thionyl chloride as previously reported literature.^18^ Finally, amide derivatives **3a–e** were synthesized by reacting the benzhydryl piperazine **4a** with the substituted cinnamic acids using EDC and HOBt methodology.^19^ The 4-chlorobenzhydryl piperazine **4b** was obtained from commercial source.

**Scheme 1:**
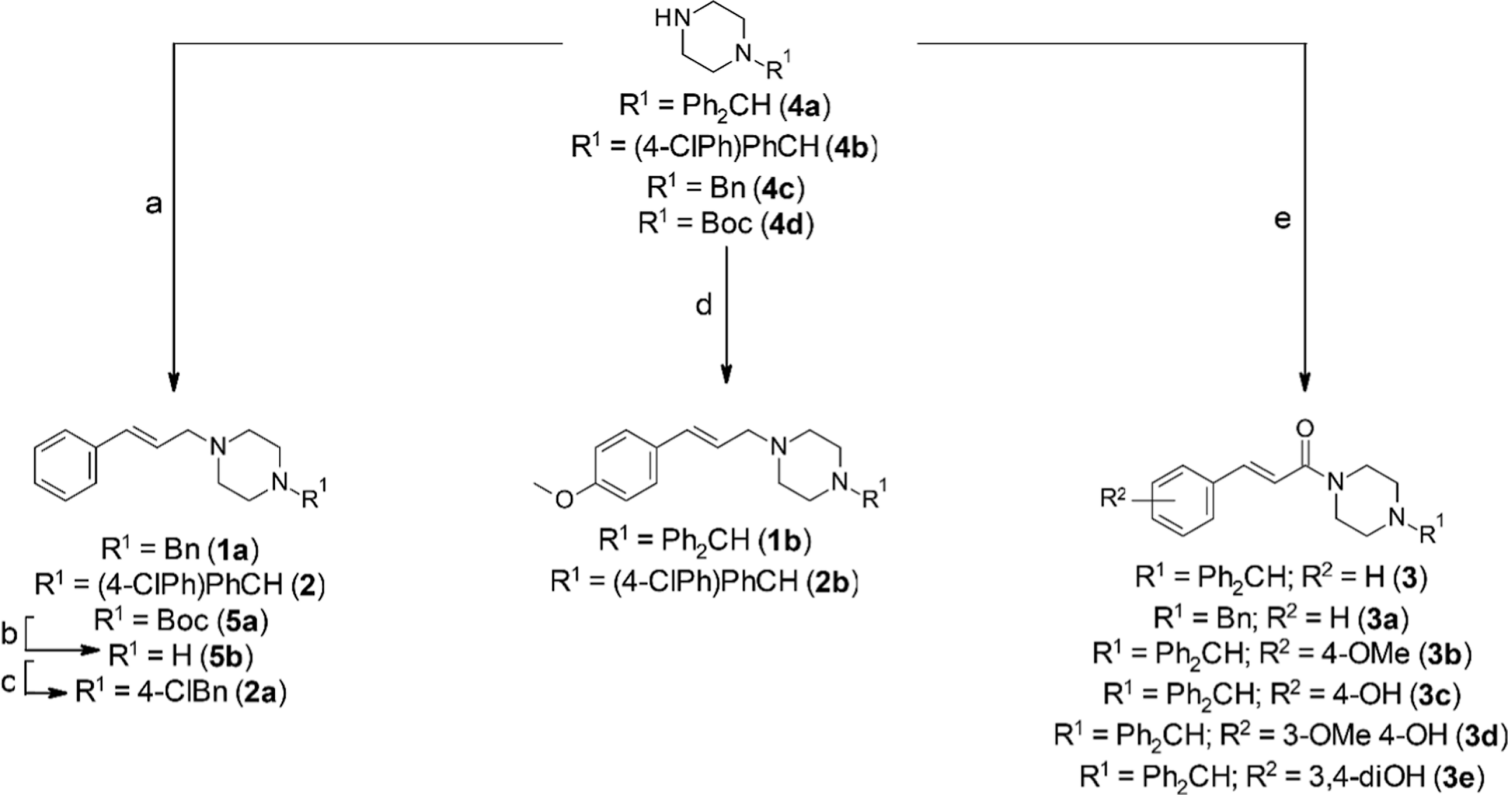
Synthetic Scheme for the Preparation of the Compounds^a^

a Reagents and conditions: (a) R^1^-Piperazines, cinnamyl chloride, K_2_CO_3_, acetone, r.t., 18-24 h; (b) TFA, DCM, 2 h; (c) 4-Chlorobenzyl chloride, K_2_CO_3_, AcN, 80 °C, 22 h; (d) Benzhydryl piperazines, 4-methoxy-cinnamaldehyde, NaBH_3_CN, EtOH, r.t., 20 h; (e) R^2^-Cinnamic acids, EDC.HCl, HOBt·*x*H_2_O, DCM or DMF then R^1^-piperazines, TEA, r.t., 18–22 h.

The compounds were tested against *A. cantonensis* first-stage (L1) and third-stage (L3) larvae to evaluate their anthelmintic potential (Figure 3 and Table 2), following the same experimental protocol previously described for cinnarizine.^11,20^ The compounds exhibited notable efficacy, with important differences in potency across the developmental stages. Clocinizine (**2**) was the most potent compound against L1 larvae with an EC_50_ of 4.2 μM, while derivatives **1b** and **2b** displayed similar activity against both stages (EC_50_ 8.2 to 9.3 μM). Compound **3a** was the more potent L1 than L3 larvae (EC_50_ 8.2 vs 18.1 μM), while cinnarizine (**1**) showed the opposite trend (EC_50_ 45.9 vs 16.3 μM).

**Figure 3:**
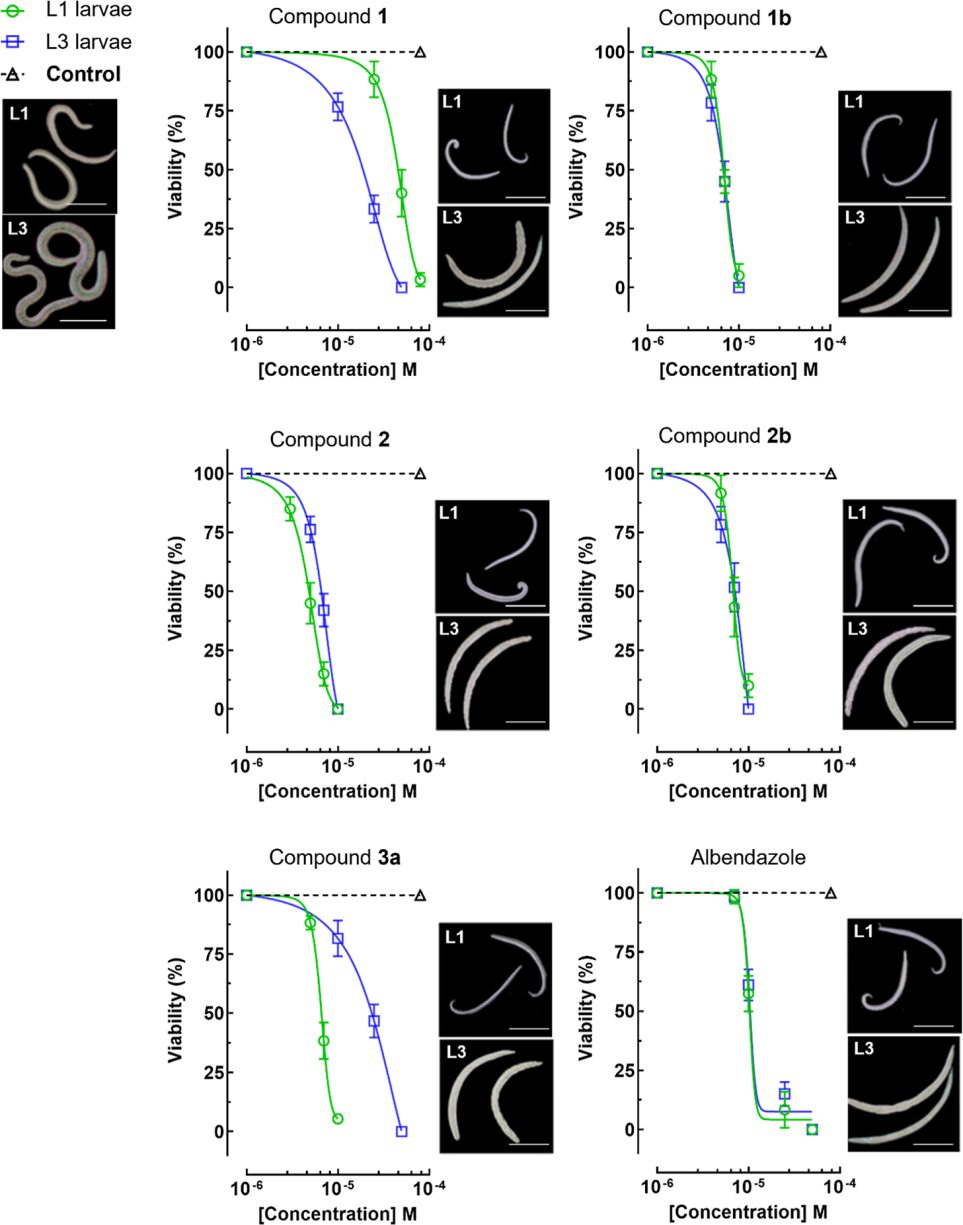
Dose-dependent effects and morphology-based phenotyping of *Angiostrongylus cantonensis* larvae exposed to cinnarizine derivatives. Left panels: dose–response curves showing larval viability after exposure to active compounds and albendazole (positive control). The dashed black line represents the untreated control. Right panels: representative light microscopy images of L1 and L3 larvae exposed to compound concentrations near their respective EC_50_ values, illustrating compound-induced morphological alterations. Left-top images: untreated control larvae. L1 larvae are excreted in the feces of the definitive host and initiate infection in the intermediate host, while L3 represent the infective stage for humans. Data are presented as mean ± standard deviation (SD) of three independent experiments.

**Table 2:** In Vitro Results for Anthelmintic Activity of Tested Compounds Against *A. cantonensis* L1 and L3 Larvae, and Toxicity Assessment Against *Caenorhabditis elegans* and Human Cells (HaCat)^a^

	*A. cantonensis* EC_50_ (μM ± SEM)			
compound	L1	L3	*C. elegans* LC_50_ (μM)	HaCat CC_50_ (μM)
**1** (cinnarizine)	45.9 ± 6.3	16.3 ± 1.8	>1000	>500
**1a**	>100	N.D.	>1000	>500
**1b**	8.2 ± 1.2	8.7 ± 0.9	>1000	>500
**2** (clocinizine)	4.2 ± 1.1	8.6 ± 1.1	>1000	>500
**2a**	>100	N.D.	>1000	>500
**2b**	9.3 ± 0.9	8.8 ± 1.2	>1000	>500
**3**	>100	N.D.	>1000	>500
**3a**	8.7 ± 1.1	18.1 ± 1.5	>1000	>500
**3b**	>100	N.D.	>1000	>500
**3c**	>100	N.D.	>1000	>500
**3d**	>100	N.D.	>1000	>500
**3e**	>100	N.D.	>1000	>500
albendazole	14.2 ± 1.1	15.6 ± 1.2	14.8 ± 1.5	>500

a N.D. not determined.

In addition to demonstrating time- and concentration-dependent effects, light microscopy was used to evaluate morphological alterations in treated larvae (Figure 3). All active compounds induced observable changes, characterized by elongated and thinner larvae compared to untreated controls. Morphological assessment of helminths exposed to anthelmintic compounds is a widely adopted strategy in the literature and contributes to the identification of phenotypic responses.^21–23^ In the context of *A. cantonensis*, previous studies have reported that antihistamines and other bioactive compounds such as piplartine produce marked morphological alterations in L1 and L3 larvae.^20^ Overall, the tested compounds were slightly more active against L1 larvae, possibly due to differences from the earlier developmental stage that makes it more susceptible to chemical insults.

To assess general toxicity and obtain a preliminary selectivity window, we performed two orthogonal counter-screens against *Caenorhabditis elegans*, a free-living nematode widely used to detect broad nematode toxicity, and against HaCaT human keratinocytes. Notably, none of the compounds affected the *C. elegans* viability and no cytotoxic effects were detected in HaCaT cells at the maximum tested concentrations. Together, these findings provide preliminary, species-specific selectivity indicators for *A. cantonensis* without establishing mammalian safety or predicting efficacy across helminths. This pattern agrees with previous reports on piplartine^20^ and organometallic compounds,^24^ which selectively targeted *A. cantonensis* without affecting *C. elegans*. Given the parasitic nature of *A. cantonensis*, this may reflect an action dependent on parasite-specific targets, uptake mechanisms or pharmacological profile.

Interestingly, the chlorinated analogues **2** and **2b** were more potent than their counterparts **1** and **1b**. In fact, the anthelmintic activity of clocinizine (**2**) was already identified by our group against *S. mansoni* in both in vitro and in vivo experiments,^15^ which is in line with the results observed here for *A. cantonensis*. Moreover, the methoxylated derivatives **1b** and **2b** were also active, being **1b** considerably more potent than cinnarizine, while the potency of **2b** against L1 larvae was comparable to **2**, suggesting that substitution in the cinnamyl ring played a role in the increased anthelmintic activity. More importantly, these methoxylated derivatives have higher solubility without important modification on lipophilicity (Table 1), representing an improvement in the drug-likeness allied to the anthelmintic activity and permeable through BBB. On the other hand, the simplified benzylpiperazine derivatives **1a** and **2a** showed no appreciable activity.

We hypothesized that the anthelmintic effect of both **1** and **2** could be correlated to their affinity on histamine receptors, since the substitution with chlorine is known to increase the antihistamine activity and selectivity of **2** at histamine H_1_ receptor over other receptors such as muscarinic receptors.^12,16^ However, literature states that in general invertebrate animals has no histaminergic neurons and do not express histamine receptors.^25^ Some studies suggest that *S. mansoni* express specific metabotropic receptors for histamine (SmGPR-1 and 2),^26^ but to date no information about histamine receptors in *A. cantonensis* was reported. It is noteworthy that compounds **1a** and **2b** were also inactive against *S. mansoni*, suggesting that both **1** and **2** may act by a different mechanism on *A. cantonensis*. Anywise, the anthelmintic activity of the compounds cannot be directly correlated to their affinity at histamine receptors.

Except for the derivative **3a**, the amide derivatives (**3** and **3b**–**e**) showed no activity against the larvae. Differently from the observed to **1b** and **2b**, the modifications in the cinnamoyl ring showed no important contribution to lethal effect to the larvae. This reinforces an independent effect of the compounds on the histamine H_1_ receptor, since the basicity of the piperazine nitrogen is important for binding at this receptor.^27,28^ Considering that compound **3a** was the only benzylpiperazine derivative that displayed activity against the larvae, the amide derivatives may have a completely different mechanism of action from the amine derivatives.

To elucidate these questions, a systematic evaluation of the structure–activity relationships of this set was performed by statistical exploratory analyses using the descriptors presented in Table 1. A principal component analysis (PCA) of the descriptors was carried out (Figure 4A), revealing that amines with anthelmintic activity (**1**, **2**, **1b** and **2b**) were grouped in the lower-right quadrant, apart from the inactive compounds. The loadings of the descriptors (see Supporting Information) indicate that presence of two basic nitrogens (represented by negative PC2 values) allied to higher lipophilicity (logP between 4.5 and 5.5) described by positive PC1 values may be related to the anthelmintic activity of benzhydryl-containing compounds. The simplified compounds **1a**, **2a** and **3a** presented negative values for PC1, describing their higher solubility and lower lipophilicity from the benzhydryl-containing compounds. Interestingly, the active benzylpiperazine **3a** was the only compound present in the upper-left quadrant, indicating a unique structural profile related to anthelmintic activity.

**Figure 4:**
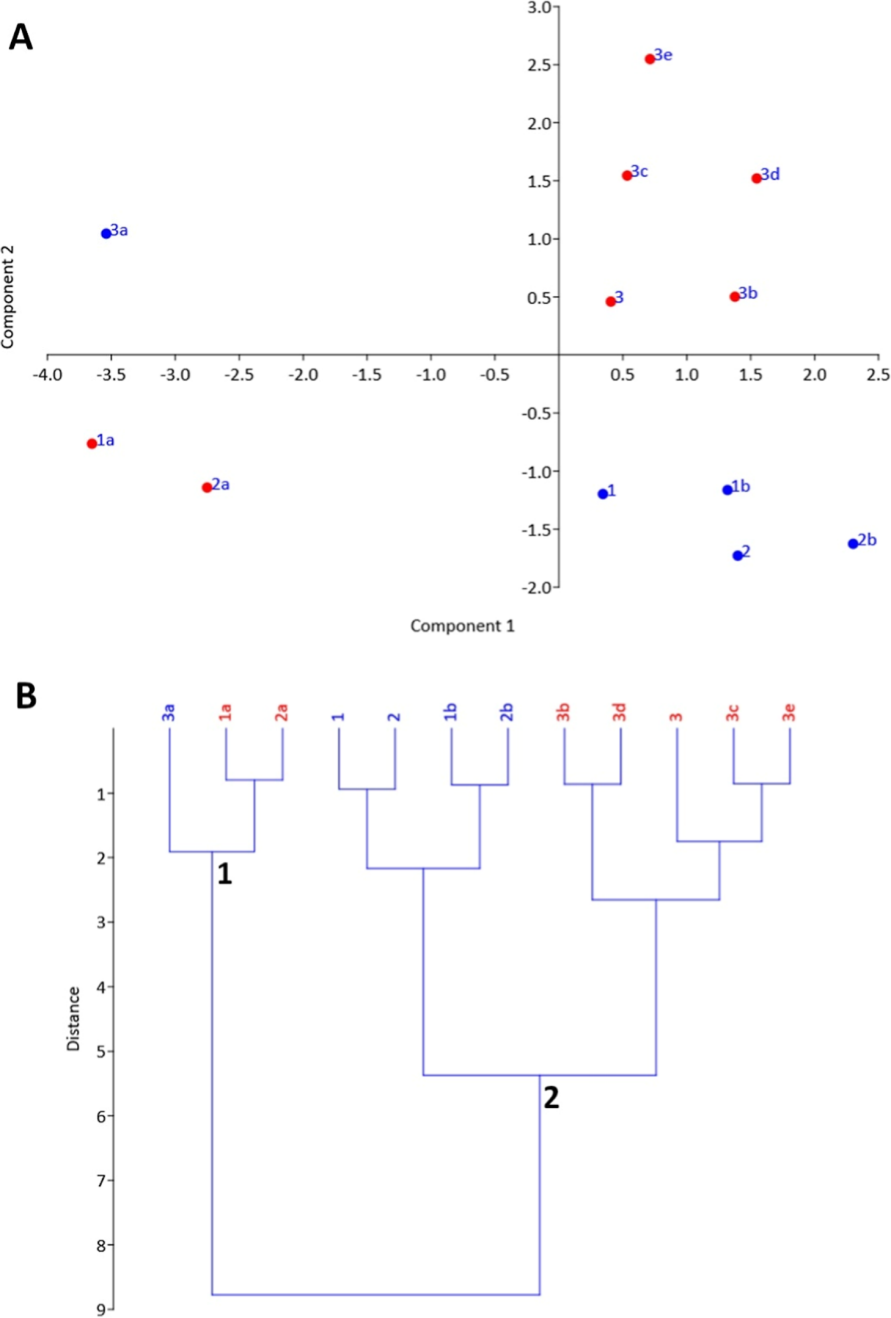
(A) Scatter plot of PC1 vs PC2 from the PCA. (B) Dendrogram obtained from the HCA, with dendron 1 containing the simplified analogues and dendron 2 the benzhydryl-containing compounds. Blue samples represent active compounds and red samples represent the inactives.

A second statistical method, the hierarchical cluster analysis (HCA) was conducted using the same descriptors, corroborating to the PCA results and reinforcing the relationship of these descriptors with the observed activity. As can be noted in Figure 4B, two main clusters were obtained, where compounds **1a**, **2a** and **3a** were clustered together in the dendron 1 (left cluster in Figure 4B) due their lower MW and higher solubility, while the benzhydryl-piperazines were grouped in the right cluster in dendron 2 (Figure 4B). In consonance with PCA results, compound **3a** presented considerable dissimilarity from the counterparts **1a** and **2a**, reinforcing its unique structural profile and suggesting a distinct mechanism of action for this compound. The active benzhydryl-piperazines **1**, **2**, **1b** and **2b** were also grouped in a separate cluster with high dissimilarity from the inactive compounds, denoting their specific properties that may be related to their activity.

In conclusion, we identified novel cinnarizine derivatives with better drug likeness and predicted BBB permeability, improved potency and in vitro efficacy against *A. cantonensis*. These compounds represent inspiring prototypes to design novel compounds with anthelmintic properties against *A. cantonensis* and hopefuly to the future treatment of neuroangiostrongyliasis. The methoxylated derivative **2b** must be highlighted due to its improved potency allied to equilibrated physicochemical properties that allow its penetration in the CNS, and compound **3a** represent a unique prototype for further modifications in the search for novel anthelmintic compounds.

## Material and Methods

### Reagents and Equipment

Chemicals were obtained in adequate purity from Sigma-Aldrich Co (Saint Louis, MO, USA) and LabSynth Co. (Diadema, Brazil) and used without any further purification. ^1^H and ^13^C NMR spectra were recorded on a Bruker Ultrashield 300 spectrometer, operating at 300 and 75 MHz, respectively, using CDCl_3_ or DMSO-*d*_6_ as solvents and tetramethylsilane (TMS) as internal standard. Chemical shifts (δ) were determined from TMS and are reported in parts per million (ppm). Coupling constants (*J*) are reported in units of Hertz (Hz), if applicable. All NMR data were obtained using the compounds as free bases. HPLC-UV analysis was performed in a LC-20AT chromatograph (Shimadzu) using a C18 column (ACE-5) and acetonitrile/H_2_O as eluent, detection at 262 nm wavelength and flow rate 1.0 mL/min. Compounds exhibiting chromatographic purity >95% were considered suitable for the anthelmintic assays. The final compounds were converted into the respective hydrogen-maleate or hydrochloride salts to increase solubility and purity.

### General Method for Synthesis of Cinnamyl Piperazines (**1a**, **2** and **5a**)

To a mixture of appropriate piperazine (0.5 mmol) and 1 mmol (0.138 g) of K_2_CO_3_ in 15 mL of acetonitrile, 0.5 mmol (0.076 g) of cinnamyl chloride were added. The reaction was stirred at 80 °C for 18–22 h. Afterward, the mixture was filtered and the solvent was evaporated under reduced pressure. The residue was taken up in 15 mL of dichloromethane (DCM) and the organic solution was washed twice with 15 mL of purified water, dried with anhydrous Na_2_SO_4_ and evaporated. The compounds were purified using a silica gel column with DCM/MeOH mixtures as the eluent.

#### 1-Benzyl-4-[(*E*)-cinnamyl]­piperazine (**1a**)

Reaction with 0.088 g of 1-benzylpiperazine (**4c**) yielded 0.076 g (52%) of **1a** as yellowish oil, mp 224–225 °C (dihydrogen-maleate salt). ^1^H NMR (300 MHz, CDCl_3_) δ: 7.51–7.12 (m, 10H), 6.50 (d, *J* = 15.9 Hz, 1H), 6.28 (dt, *J* = 15.8, 6.7 Hz, 1H), 3.51 (s, 2H), 3.15 (d, *J* = 6.7 Hz, 2H), 2.51 (br s, 8H). ^13^C NMR (75 MHz, CDCl_3_) δ: 138.2, 137.0, 133.0, 129.2, 128.6, 128.2, 127.5, 127.1, 126.7, 126.4, 63.1, 61.1, 53.3, 53.1. Spectroscopic data is in accordance with literature.^15,29^

#### 1-[(4-Chlorophenyl)-phenyl-methyl]-4-[(*E*)-cinnamyl]­piperazine (**2**)

Reaction with 0.143 g of 1-(4-chlorobenzhydryl)­piperazine (**4b**) yielded 0.111 g (55%) of **2** as white solid, mp 163–164 °C (dihydrogen-maleate salt). ^1^H NMR (300 MHz, CDCl_3_) δ: 7.54–6.98 (m, 14H), 6.51 (d, *J* = 15.9 Hz, 1H), 6.36–6.14 (m, 1H), 4.22 (s, 1H), 3.17 (d, *J* = 6.7 Hz, 2H), 2.54 (br s, 4H), 2.43 (br s, 4H). ^13^C NMR (75 MHz, CDCl_3_) δ: 142.1, 141.4, 136.9, 133.1, 132.5, 129.2, 128.6, 128.6, 128.5, 127.9, 127.5, 127.1, 126.4, 126.3, 75.4, 60.9, 53.4, 51.7. Spectroscopic data is in accordance with literature.^15,30^ *tert*-Butyl-4-[(*E*)-cinnamyl]­piperazine-1-carboxylate (**5a**). Reaction with 0.093 g of 1-*tert*-butyloxypiperazine (**4d**) yielded 75% (0.113 g) of **5a** as yellowish oil. ^1^H NMR (300 MHz, CDCl_3_) δ: 7.45–7.13 (m, 5H), 6.52 (d, *J* = 15.9 Hz, 1H), 6.24 (dt, *J* = 15.8, 6.7 Hz, 1H), 3.50–3.40 (m, 4H), 3.16 (d, *J* = 6.7 Hz, 2H), 2.50–2.40 (m, 4H), 1.46 (s, 9H). Spectroscopic data is in accordance with literature.^31^

### Synthesis of 4-[(*E*)-cinnamyl]­piperazine (**5b**)

Compound **5b** was prepared as described by Sweeney et al. (2018).^31^ Briefly, compound **5a** was dissolved in 10 mL of DCM and 2 mL of trifluoroacetic acid (TFA) was added and stirred at room temperature for 2 h. The organic solution was washed with 3 × 15 mL aqueous NaHCO_3_ solution, and the organic layer was dried with anhydrous Na_2_SO_4_ and evaporated. Compound **5b** was obtained with 75% yield (0.061 g). ^1^H NMR (300 MHz, CDCl_3_) δ: 7.46–7.12 (m, 5H), 6.52 (d, *J* = 15.9 Hz, 1H), 6.34–6.17 (m, 1H), 3.14 (d, *J* = 6.7 Hz, 2H), 2.96–2.84 (m, 4H), 2.48 (br s, 4H), 2.08 (s, 2H). Spectroscopic data is in accordance with literature.^31^

### Synthesis of 1-[(4-Chlorophenyl)­methyl]-4-[(*E*)-cinnamyl]­piperazine (**2a**)

To a mixture of 0.3 mmol of **5b** (0.061 g) and 0.6 mmol (0.082 g) of K_2_CO_3_ in 15 mL of acetonitrile, 0.3 mmol (0.048 g) of 4-chlorobenzyl chloride were added. The reaction was stirred at 80 °C for 18–22 h. Afterward, the mixture was filtered and the solvent was evaporated under reduced pressure. The residue was taken up in 15 mL of dichloromethane (DCM) and the organic solution was washed with 2 × 15 mL of purified water, dried with anhydrous Na_2_SO_4_ and evaporated. The crude material was purified using a silica gel column with DCM/MeOH (20:1) as eluent to give **2a** with 78% yield (0.075 g) as yellowish oil, mp 287–288 °C (dihydrochloride salt). ^1^H NMR (300 MHz, CDCl_3_) δ: 7.47–7.11 (m, 9H), 6.52 (d, *J* = 15.8 Hz, 1H), 6.27 (dt, *J* = 15.8, 6.8 Hz, 1H), 3.48 (s, 2H), 3.17 (d, *J* = 6.8 Hz, 2H), 2.50 (br s, 8H). ^13^C NMR (75 MHz, CDCl_3_) δ: 136.9, 136.8, 133.1, 132.7, 130.4, 128.6, 128.4, 127.5, 126.6, 126.3, 62.2, 61.0, 53.2, 53.0. Spectroscopic data is in accordance with literature.^15^

### Synthesis of Benzhydryl Piperazine (**4a**)

To a solution of 5 mmol (0.92 g) of benzhydrol in 20 mL of DCM, 1.5 mL of thionyl chloride was added and the mixture was heated to 60 °C for 12 h. Afterward the organic solution was washed with 2 × 20 mL of saturated NaHCO_3_ solution, 20 mL of water and then dried over anhydrous Na_2_SO_4_. The organic phase and the excess of thionyl chloride were evaporated. The residue was redissolved in 20 mL of acetonitrile, and 15 mmol (1.29 g) of piperazine and 5 mmol (0.70 g) of K_2_CO_3_ were added and the reaction mixture was stirred at 80 °C for 18 h. After cooling down, mixture was filtered, the solvent was evaporated and the residue was taken up in 20 mL of HCl solution (pH 1–2) and washed with 3 × 10 mL of ethyl acetate. The aqueous phase was then alkalinized to pH 10–11 with NaOH solution and extracted with 3 × 10 mL of EtOAc. The organic layer was dried with anhydrous Na_2_SO_4_ and evaporated to give benzhydryl piperazine **4a** (0.75 g, 60% yield) with adequate purity for the next reactional steps as colorless oil. ^1^H NMR (300 MHz, CDCl_3_) δ: 7.46–7.33 (m, 4H), 7.31–7.19 (m, 4H), 7.15 (dd, *J* = 7.2, 1.2 Hz, 2H), 4.21 (s, 1H), 2.94 (s, 1H), 2.91–2.82 (m, 4H), 2.37 (br s, 4H). Spectroscopic data is in accordance with literature.^18^

### Synthesis of Benzyl Piperazine (**4c**)

To a solution of 2 mmol (0.244 g) of piperazine monohydrochloride in 20 mL of EtOH, 1 mmol (0.126 g) of benzyl chloride was added and stirred at 80 °C for 2 h. The obtained suspension was filtered and the solvent was evaporated under reduced pressure. The oily residue was dissolved in 15 mL of water and washed with 2 × 10 mL of EtOAc, and the aqueous solution was alkalinized to pH 10–11 and extracted with 3 × 10 mL of EtOAc. The organic phase was dried with anhydrous Na_2_SO_4_ and evaporated to give **4c** with 60% yield (0.106 g) and adequate purity to the next reactional step. ^1^H NMR (300 MHz, CDCl_3_) δ: 7.34–7.26 (m, 5H), 3.55 (s, 2H), 3.25 (br s, 4H), 2.80 (br s, 4H). Spectroscopic data is in accordance with literature.^32^

### General Preparation of 4-Methoxycinnamyl Piperazines **1b** and **2b**

To a solution of 0.75 mmol (0.121 g) of 4-methoxycinnamaldehyde and 0.5 mmol of benzhydryl piperazines (**4a** or **4b**) in 15 mL of ethanol, 1.5 mmol (0.094 g) of sodium cyanoborohydride were added. The reaction mixture was stirred at room temperature for 20 h, when the solvent was evaporated and the residue was taken up in 15 mL of ethyl acetate. The organic phase was washed with 2 × 15 mL of aqueous saturated NaHCO_3_ solution, 15 mL of purified water and dried with anhydrous Na_2_SO_4_. The crude product was purified through silica gel column chromatography using DCM/MeOH (100 to 20:1) as eluent.

#### 1-Benzhydryl-4-[(*E*)-3-(4-methoxyphenyl)­allyl]­piperazine (**1b**)

Reaction with **4a** (0.126 g) yielded 0.082 g (41%) of **1b** as white solid, mp 172–174 °C (dihydrogen-maleate salt). ^1^H NMR (300 MHz, CDCl_3_) δ: 7.40 (d, *J* = 7.5 Hz, 4H), 7.34–7.20 (m, 6H), 7.16 (t, *J* = 7.2 Hz, 2H), 6.83 (d, *J* = 8.5 Hz, 2H), 6.44 (d, *J* = 15.9 Hz, 1H), 6.22–5.98 (m, 1H), 4.23 (s, 1H), 3.79 (s, 3H), 3.14 (d, *J* = 6.7 Hz, 2H), 2.53 (br s, 4H), 2.45 (br s, 4H). ^13^C NMR (75 MHz, CDCl_3_) δ: 160.4, 141.5, 139.7, 128.8, 128.5, 127.6, 127.5, 127.4, 114.2, 113.9, 75.1, 59.4, 55.3, 51.6, 48.5.

#### 1-[(4-Chlorophenyl)-phenyl-methyl]-4-[(*E*)-3-(4-methoxyphenyl)­allyl]­piperazine (**2b**)

Reaction with **4b** (1.143 g) yielded 0.076 g (35%) of **2b** as white solid, mp 169–170 °C (dihydrogen-maleate salt). ^1^H NMR (300 MHz, CDCl_3_) δ: 7.37–7.16 (m, 11H), 6.83 (d, *J* = 8.6 Hz, 2H), 6.44 (d, *J* = 15.8 Hz, 1H), 6.20–5.99 (m, 1H), 4.21 (s, 1H), 3.79 (s, 3H), 3.14 (d, *J* = 6.8 Hz, 2H), 2.54 (br s, 4H), 2.43 (br s, 4H). ^13^C NMR (75 MHz, CDCl_3_) δ: 159.2, 142.2, 141.4, 132.7, 132.5, 129.7, 129.2, 128.6, 128.5, 127.9, 127.5, 127.2, 123.9, 114.0, 75.4, 61.1, 55.3, 53.3, 51.7.

### General Preparation of Cinnamoyl Piperazines **3**

To a solution of 0.2 mmol appropriate cinnamic acid in 10 mL of DCM (for cinnamic, 4-methoxycinnamic and ferulic acids) or DMF (for 4-coumaric and caffeic acids), 0.2 mmol (0.038 g) of 1-ethyl-3-(3-(dimethylamino)­propyl)­carbodiimide hydrochloride (EDC·HCl) and 0.2 mmol (0.030 g) of hydroxybenzotriazole hydrate (HOBt.*x*H_2_O) were added and stirred at room temperature for 1 h. Afterward, a solution of 0.22 mmol of **4a** (0.055 g) or **4c** (0.038 g) in 10 mL of DCM or DMF was added and reacted for 18–22 h. The organic mixture was evaporated, taken up in DCM and washed with 2 × 10 mL of saturated NaHCO_3_ solution, 10 mL of water and dried with anhydrous Na_2_SO_4_. The solvent was evaporated, and the residue was purified through flash chromatography using DCM/MeOH (20:1) as eluent.

#### (*E*)-1-(4-Benzhydrylpiperazin-1-yl)-3-phenyl-prop-2-en-1-one (**3**)

Reaction of 0.030 g of cinnamic acid with **4a** yielded 0.063 g (85%) of **3** as white solid, mp 154–155 °C (hydrochloride salt). ^1^H NMR (300 MHz, CDCl_3_) δ: 7.64 (d, *J* = 15.4 Hz, 1H), 7.48 (dd, *J* = 7.2, 2.1 Hz, 2H), 7.42 (d, *J* = 7.1 Hz, 4H), 7.38–7.30 (m, 4H), 7.31–7.27 (m, 3H), 7.21 (d, *J* = 7.2 Hz, 2H), 6.83 (d, *J* = 15.4 Hz, 1H), 4.26 (s, 1H), 3.74 (br s, 2H), 3.66 (br s, 2H), 2.52–2.35 (m, 4H). ^13^C NMR (75 MHz, CDCl_3_) δ: 165.3, 142.6, 142.1, 135.3, 129.6, 128.8, 128.6, 127.9, 127.7, 127.2, 117.1, 75.9, 52.2, 51.6, 46.0, 42.4.

#### (*E*)-1-(4-Benzylpiperazin-1-yl)-3-phenyl-prop-2-en-1-one (**3a**)

Reaction of 0.030 g of cinnamic acid with **4a** yielded 0.040 g (60%) of **3a** as colorless oil, mp 193–194 °C (hydrogen-maleate salt). ^1^H NMR (300 MHz, CDCl_3_) δ: 7.66 (d, *J* = 15.4 Hz, 1H), 7.57–7.43 (m, *J* = 5.2, 2.7 Hz, 2H), 7.43–7.14 (m, 8H), 6.86 (d, *J* = 15.4 Hz, 1H), 3.74 (br s, 2H), 3.64 (br s, 2H), 3.53 (s, 2H), 2.54–2.37 (m, 4H). ^13^C NMR (75 MHz, CDCl_3_) δ: 165.4, 142.7, 137.6, 135.3, 129.6, 129.2, 128.8, 128.4, 127.8, 127.3, 117.2, 62.9, 53.3, 52.8, 45.9, 42.2.

#### (*E*)-1-(4-Benzhydrylpiperazin-1-yl)-3-(4-methoxyphenyl)­prop-2-en-1-one (**3b**)

Reaction of 0.036 g of 4-methoxycinnamic acid with **4a** yielded 0.058 g (72%) of **3b** as white solid, mp 113–114 °C (hydrochloride salt). ^1^H NMR (300 MHz, CDCl_3_) δ: 7.62 (d, *J* = 15.3 Hz, 1H), 7.42 (dd, *J* = 8.1, 4.2 Hz, 6H), 7.28 (t, *J* = 7.4 Hz, 4H), 7.19 (t, *J* = 7.2 Hz, 2H), 6.87 (d, *J* = 8.6 Hz, 2H), 6.70 (d, *J* = 15.4 Hz, 1H), 4.25 (s, 1H), 3.81 (s, 3H), 3.72 (br s, 2H), 3.64 (br s, 2H), 2.48–2.33 (m, 4H). ^13^C NMR (75 MHz, CDCl_3_) δ: 165.6, 160.8, 142.4, 142.2, 129.3, 128.6, 128.1, 127.9, 127.2, 114.6, 114.2, 75.9, 55.4, 52.2, 51.8, 45.8, 42.2.

#### (*E*)-1-(4-Benzhydrylpiperazin-1-yl)-3-(4-hydroxyphenyl)­prop-2-en-1-one (**3c**)

Reaction of 0.032 g of 4-coumaric acid with **4a** yielded 0.056 g (70%) of **3d** as yellowish solid, mp 201–202 °C (hydrochloride salt). ^1^H NMR (300 MHz, DMSO) δ: 9.80 (br s, 1H), 7.53–7.38 (m, 6H), 7.32 (dd, *J* = 15.2, 7.9 Hz, 4H), 7.25–7.10 (m, 2H), 6.95 (d, *J* = 15.3 Hz, 1H), 6.75 (d, *J* = 8.2 Hz, 2H), 4.34 (s, 1H), 3.67 (br s, 2H), 3.59 (br s, 2H), 2.31 (br s, 4H). ^13^C NMR (75 MHz, DMSO) δ: 165.1, 159.4, 142.9, 142.2, 130.1, 129.0, 128.1, 127.4, 116.0, 114.7, 75.2.

#### (*E*)-1-(4-Benzhydrylpiperazin-1-yl)-3-(4-hydroxy-3-methoxy-phenyl)­prop-2-en-1-one (**3d**)

Reaction of 0.038 g of ferulic acid with **4a** yielded 0.060 g (70%) of **3d** as yellowish solid, mp 192–193 °C (hydrochloride salt). ^1^H NMR (300 MHz, CDCl_3_) δ: 7.58 (d, *J* = 15.3 Hz, 1H), 7.42 (d, *J* = 7.3 Hz, 4H), 7.33–7.24 (m, 5H), 7.20 (t, *J* = 7.1 Hz, 2H), 7.05 (d, *J* = 8.2 Hz, 1H), 6.96 (s, 1H), 6.89 (d, *J* = 8.2 Hz, 1H), 6.67 (d, *J* = 15.3 Hz, 1H), 4.26 (s, 1H), 3.90 (s, 3H), 3.71 (br s, 2H), 3.66 (br s, 2H), 2.49–2.36 (m, 4H). ^13^C NMR (75 MHz, CDCl_3_) δ: 165.6, 147.3, 146.6, 142.8, 142.2, 128.6, 127.9, 127.2, 121.9, 114.7, 114.5, 109.7, 75.9, 55.9, 52.2, 51.8.

#### (*E*)-1-(4-Benzhydrylpiperazin-1-yl)-3-(3,4-dihydroxyphenyl)­prop-2-en-1-one (**3e**)

Reaction of 0.037 g of caffeic acid with **4a** yielded 0.053 g (65%) of **3e** as yellowish solid, mp 180–181 °C (hydrochloride salt). ^1^H NMR (300 MHz, DMSO-*d*_6_) δ: 9.41 (br s, 1H), 8.95 (br s, 1H), 7.45 (d, *J* = 7.2 Hz, 4H), 7.37–7.25 (m, 5H), 7.20 (t, *J* = 7.3 Hz, 2H), 7.04 (d, *J* = 1.9 Hz, 1H), 6.95 (dd, *J* = 8.2, 1.9 Hz, 1H), 6.87 (d, *J* = 15.3 Hz, 1H), 6.73 (d, *J* = 8.1 Hz, 1H), 4.35 (s, 1H), 3.67 (br s, 2H), 3.59 (br s, 2H), 2.31 (br s, 4H). ^13^C NMR (75 MHz, DMSO-*d*_6_) δ: 165.1, 147.8, 145.9, 142.9, 142.6, 129.0, 128.1, 127.4, 127.2, 121.1, 116.0, 115.3, 114.7, 75.2.

### In silico Analysis

The in silico analysis was carried out in the SwissADME online platform^17^ to calculate the descriptors from Table 1. From the retrieved descriptors, molecular weight (MW), fraction of sp3 carbons (Fsp3), rotatable bonds (RB) topological polar surface area (TPSA), molar refractivity (MR), consensus partition coefficient (logP—the average value from the five different methods available in the platform), water solubility (logS—ESOL topological method) were used in the statistical analysis described in the following section.

The estimation of the gastrointestinal tract (GIT) absorption and BBB permeation was done using the BOILED-Egg model^33^ implemented in the platform, using the Wiener logP (WlogP) and the TPSA values.

### Statistical Analysis

The calculated descriptors from Table 1 were employed in the PCA and HCA presented in Figure 4. For that, the crude values were normalized by autoscaling process and used in the analyses. The analyses were carried out in the Past software (version 4.13).^34^ For PCA, the given descriptors produced two main principal components (PC1 and PC2) that accumulate 92.0% of the total variance of the descriptors (PC1 = 62.1% and PC2 = 29.0%). The loading values for each descriptor are presented in Table S1, and used to obtain the scatter plot presented in Figure 4A. These descriptors were also employed in the HCA by using the Ward’s method implemented in the software. This method uses the Euclidean distances of the samples to build the dendrogram presented in Figure 4B.

### Parasite and Animal Maintenance

The *A. cantonensis* (NPDN-AC strain) life cycle was sustained in laboratory conditions at the Research Center on Neglected Diseases, Guarulhos University. Definitive hosts (Wistar rats, *Rattus norvegicus*) and intermediate hosts (freshwater snails *Biomphalaria glabrata* or giant African land snails *Achatina fulica*) were housed in controlled environments (22 ± 1 °C, 50–60% humidity) with unrestricted access to food and water. The free-living nematode *C. elegans* (Bristol N2 strain) was cultured on nematode growth medium (NGM) agar plates seeded with *Escherichia coli* OP5047 as a nutrient source.^35^

### Anthelmintic Activity

The L1 larvae were collected from fecal samples of infected Wistar rats using the Rugai sedimentation method.^36^ Larvae were washed three times in RPMI 1640 medium supplemented with 100 U/mL penicillin and 100 μg/mL streptomycin. Approximately 50 larvae were allocated into individual wells of a 96-well plate containing 200 μL of final volume of RPMI medium.^37^ For infective L3 testing, larvae were harvested from experimentally infected *A. fulica* snails through pepsin-HCl digestion (1% pepsin in 0.7% HCl, 37 °C, 2 h),^38^ followed by Rugai sedimentation. Approximately 50 larvae were transferred to each well of a 96-well plate containing RPMI medium.

Test compounds were dissolved in DMSO (final concentration ≤0.5%) and serially diluted starting from 100 μM. Plates were incubated at 21 °C for 24 h, and larval motility was assessed microscopically in an inverted microscope (100× magnification) using a four-tiered classification system: immobile, intermittent, slow, or highly active.^11^ A compound was deemed effective if ≥ 60% of larvae showed complete immobility. Larval viability was monitored at 0 and 24 h post-treatment. The efficacy of the compounds was determined by nonlinear regression as the concentration to reduce 50% of the viability (EC_50_) of the larvae. Assays were performed in triplicate and repeated three times.

### Toxicity to *C. elegans*

Synchronized fourth-stage (L4) *C. elegans* larvae were distributed into 96-well plates containing M9 buffer (60 larvae/well). Test compounds were evaluated at concentrations up to 1000 μM, with albendazole as the positive control and 0.5% DMSO as the vehicle control.^39^ After 24 h incubation at 21 °C, larval survival was determined by motility: larvae unresponsive to gentle mechanical stimulation were classified as nonviable. Toxicity was defined as ≥ 60% immobility, and the efficacy was determined by nonlinear regression as the concentration to reduce 50% of the viability (EC_50_) of the larvae.^24^ Assays were performed in triplicate and repeated three times.

### Cytotoxicity Assays

HaCat cells were maintained in DMEM medium supplemented with 10% fetal bovine serum, penicillin (100 U/mL), and streptomycin (100 μg/mL) at 37 °C with 5% CO_2_. The cells were seeded into 96-well plates at 2 × 10^3^ cells per well and incubated with the test compounds at concentrations starting from 500 μM, with 0.5% DMSO as the negative control. Cytotoxicity was assessed using the MTT assay. After 72 h, MTT solution was added, followed by 3 h of incubation. Absorbance was measured at 595 nm using an Epoch Microplate Spectrophotometer (BioTek Instruments, Winooski, VT, USA). Assays were performed in triplicate and repeated three times. Cytotoxicity was estimated through nonlinear regression as the ratio of the 50% cytotoxic concentration (CC_50_) in the cells.^24^

Ethical Statement All experimental procedures involving animals were approved by the Committee for the Ethical Use of Animals in Experimentation at Guarulhos University (Guarulhos, SP, Brazil), under protocol number 064/24. The study was conducted in full compliance with Brazilian legislation on the care and use of laboratory animals and adhered to all applicable ethical guidelines.

## Supplementary Material



## Notes

The copies of ^1^H and ^13^C NMR spectra and supplementary table S1 containing the descriptors’ loadings from the PCA (PDF)

## Abbreviations

BBB: blood–brain barrier
DCM: dichloromethane
DMEM: Dulbecco’s modified Eagle medium
DMF: *N*,*N*-dimethylformamide
DMSO: dimethyl sulfoxide
EDC·HCl: 1-ethyl-3-(3-(dimethylamino)­propyl)­carbodiimide hydrochloride
Fsp3: fraction of sp3 carbons
GIT: gastrointestinal tract
HCA: hierarchical cluster analysis
HOBt: hydroxybenzotriazole
L1: first-stage larvae
L3: third-stage larvae
logP: calculated *n*-octanol–water partition coefficient
logS: calculated water solubility
mp: melting point range
MR: molar refractivity
MTT: 3-(4,5-dimethylthiazol-2-yl)-2,5-diphenyltetrazolium bromide
MW: molecular weight
NMR: nuclear magnetic resonance
PC: principal component
PCA: principal component analysis
RB: rotatable bonds
TFA: trifluoroacetic acid
TPSA: topological polar surface area
WlogP: Wiener logP
