# Supplementary material for: Structural Exploitation of Cinnarizine Identified Novel Drug-Like Anthelmintic Agents Against Angiostrongylus cantonensis
Source: ACS Infect Dis. 2025 Oct 1;11(10):2871–81. doi: 10.1021/acsinfecdis.5c00634 (PMC12519461; doi:10.1021/acsinfecdis.5c00634)
Supplement: Supplementary file 1 [file id5c00634_si_001.pdf]

Structural exploitation of cinnarizine identified novel drug-like anthelmintic agents against *Angiostrongylus cantonensis*

Bruna L. Lemes,[1] *Mariana A. Siegl-Breno,*[2] *Mikaelly K. Silva-Nunes,*[1,3] *Flavia B. Lopes,*[2,4] *Aline S. Silva,*[3] *Natalia E. P. Motta,*[2] *Josué de Moraes,*[1,3]* *João Paulo S. Fernandes*[2]*

[1]Research Center on Neglected Diseases, Guarulhos University, Guarulhos-SP, Brazil

[2]Department of Pharmaceutical Sciences, Federal University of São Paulo, Diadema-SP, Brazil

[3]Research Center on Neglected Diseases, Scientific and Technological Institute, Brasil University, São Paulo-SP, Brazil

[4]Department of Medicine, Federal University of São Paulo, São Paulo-SP, Brazil

*Corresponding authors. moraesnpdn@gmail.com; joao.fernandes@unifesp.br

Supporting Information

Copies of the NMR spectra of the tested compounds S2-S12

Supplementary Table S1 .... S13

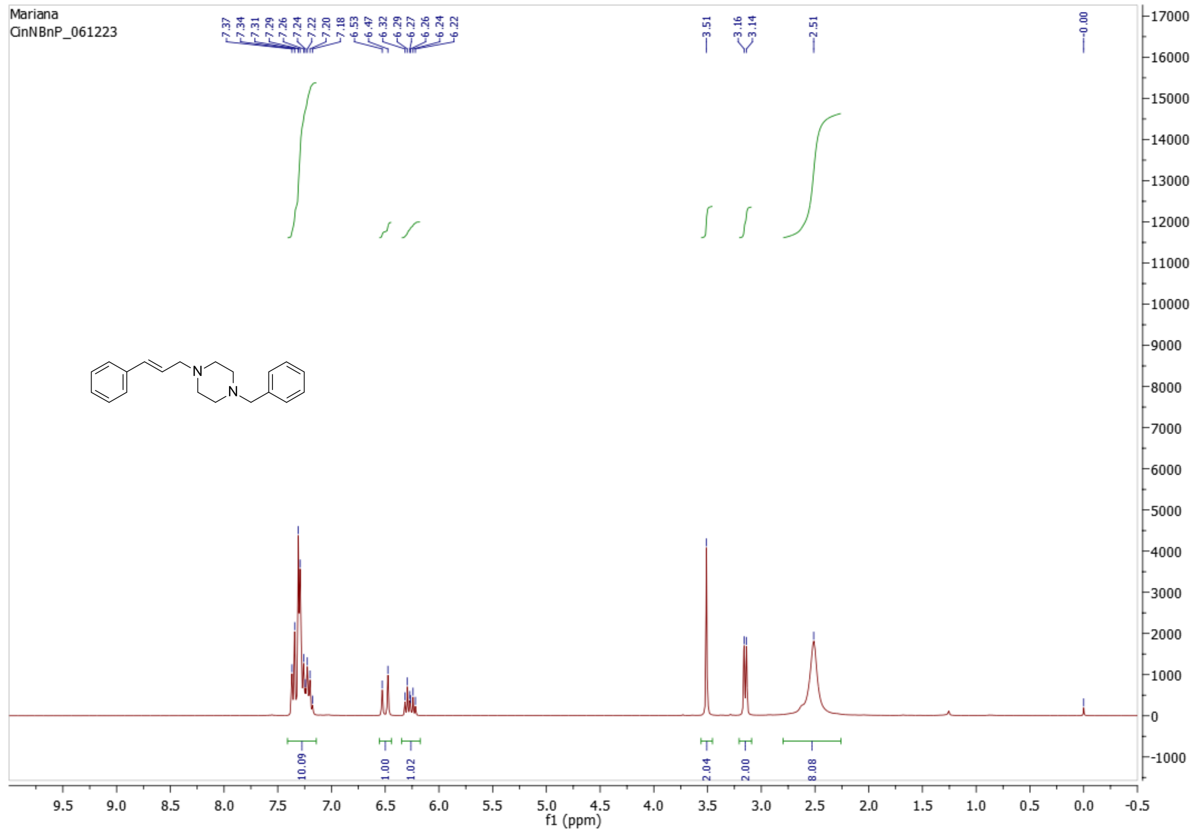

Figure S1. ^{1}H-NMR spectrum of compound **1a**

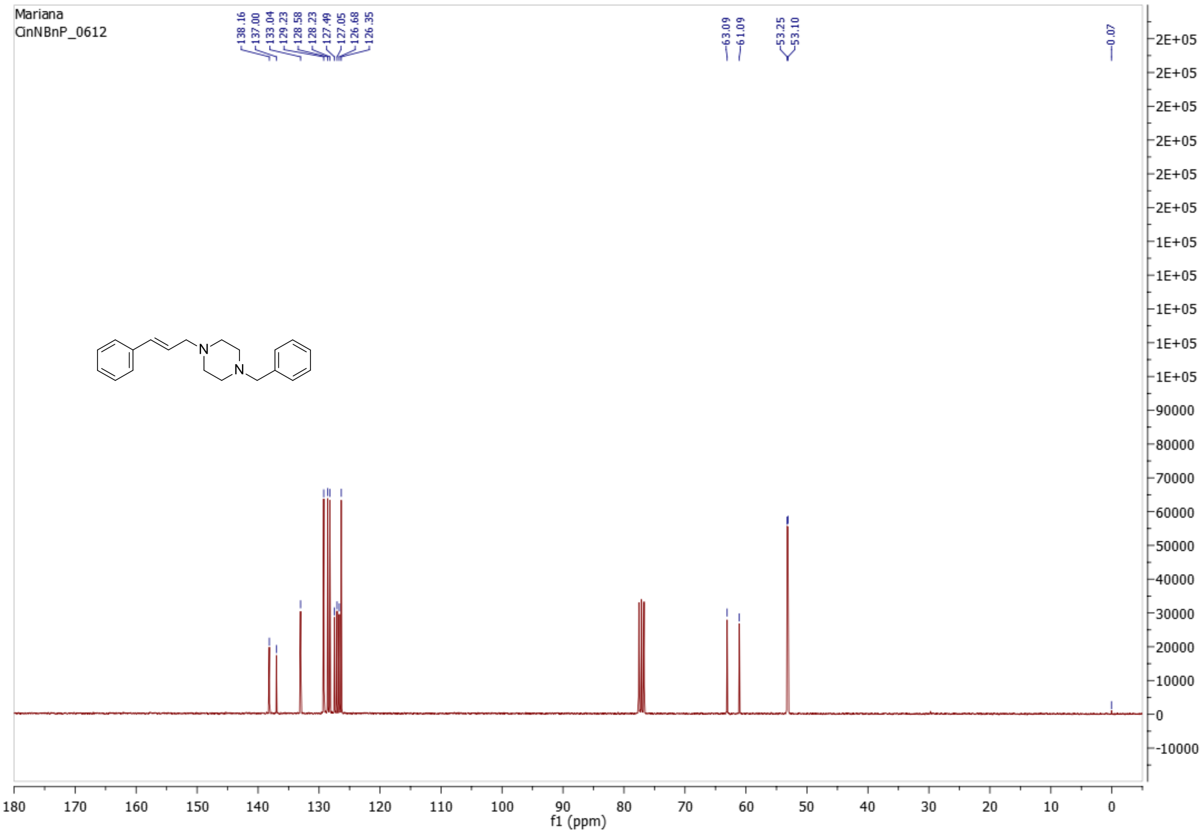

Figure S2. ^{13}C-NMR spectrum of compound **1a**

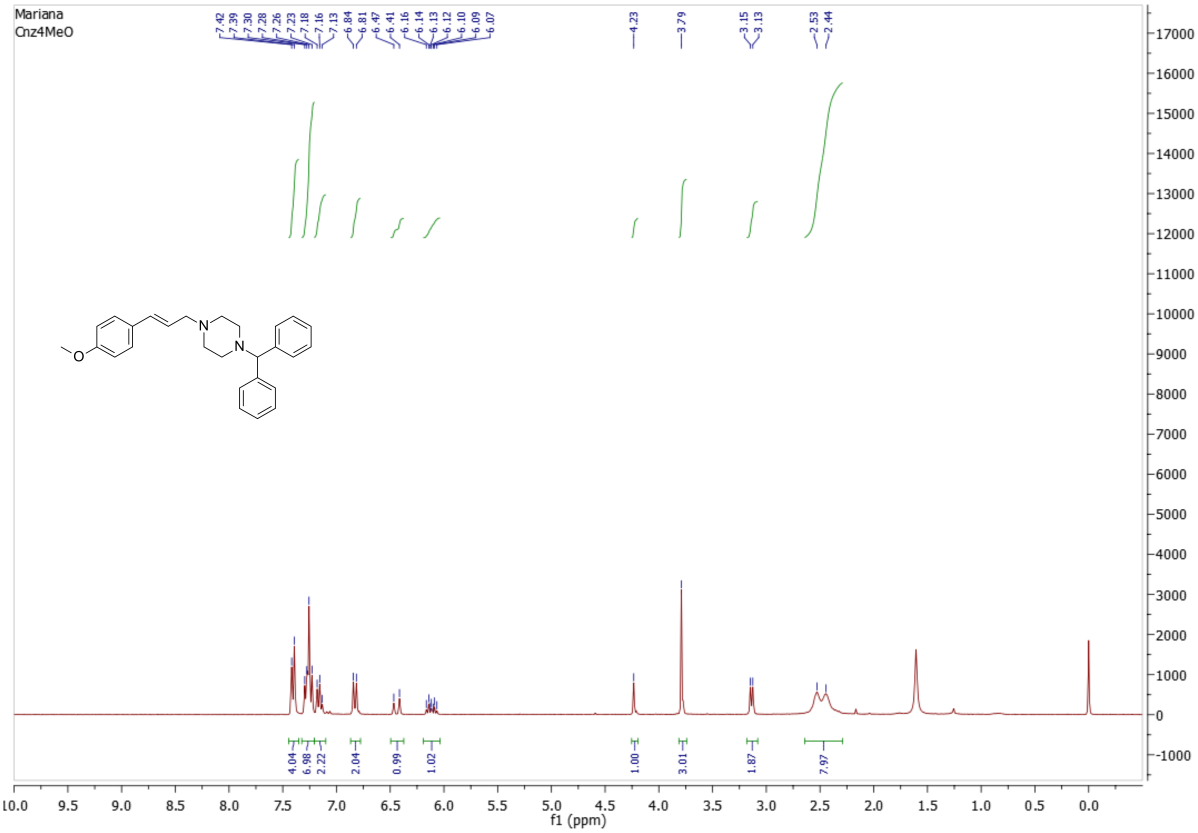

Figure S3. ^{1}H-NMR spectrum of compound **1b**

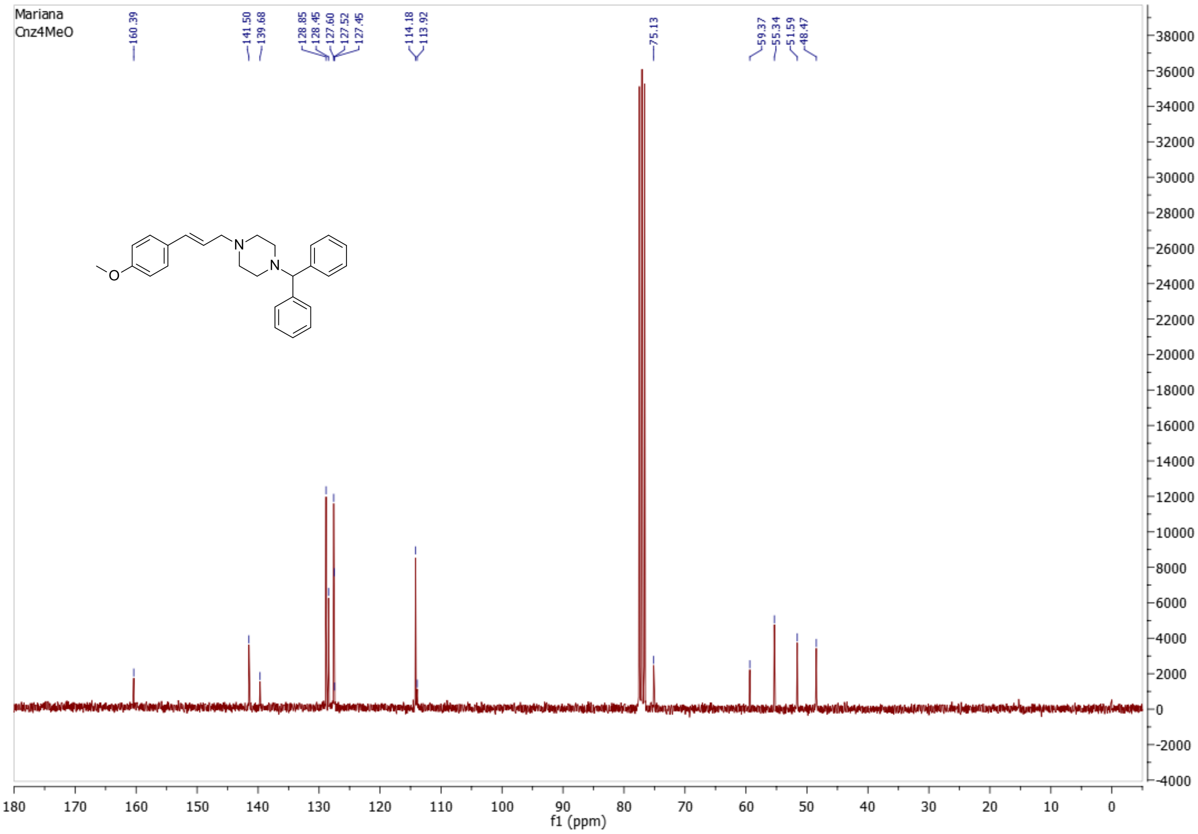

Figure S4. ^{13}C-NMR spectrum of compound **1b**

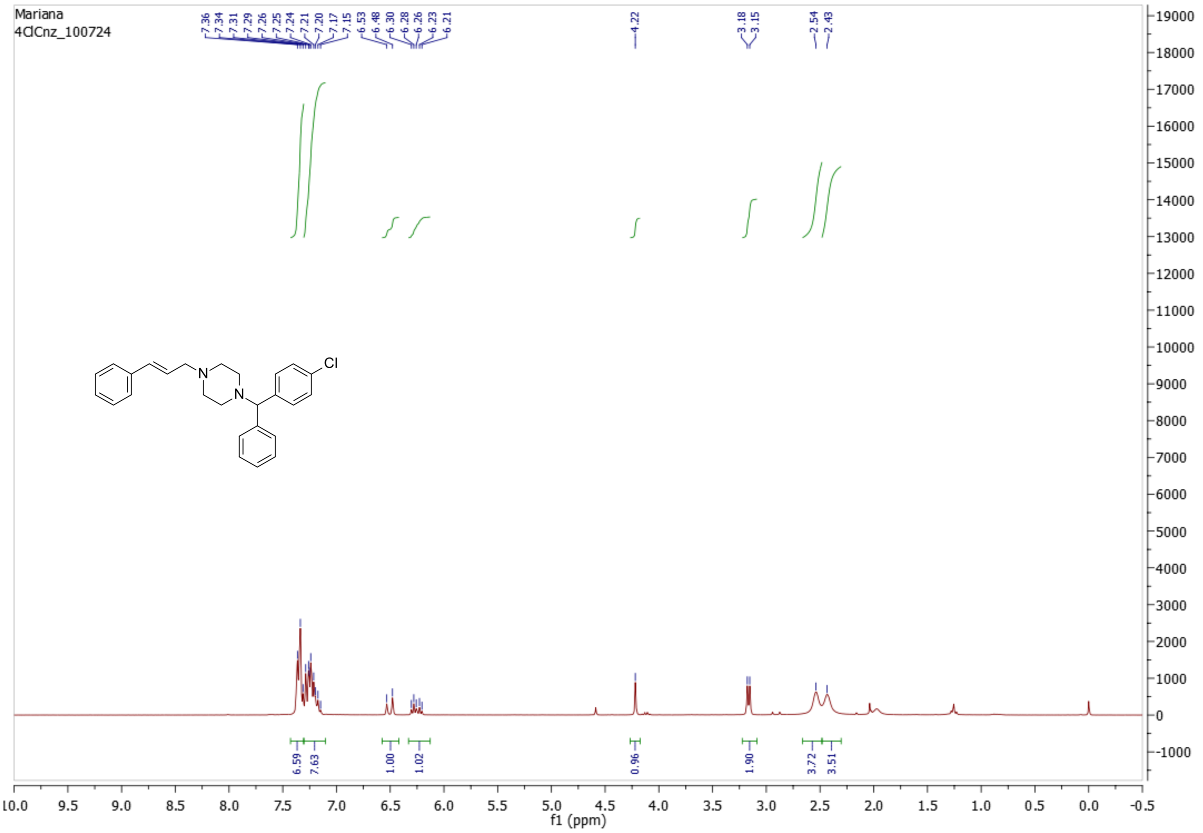

Figure S5. ^{1}H-NMR spectrum of compound **2**

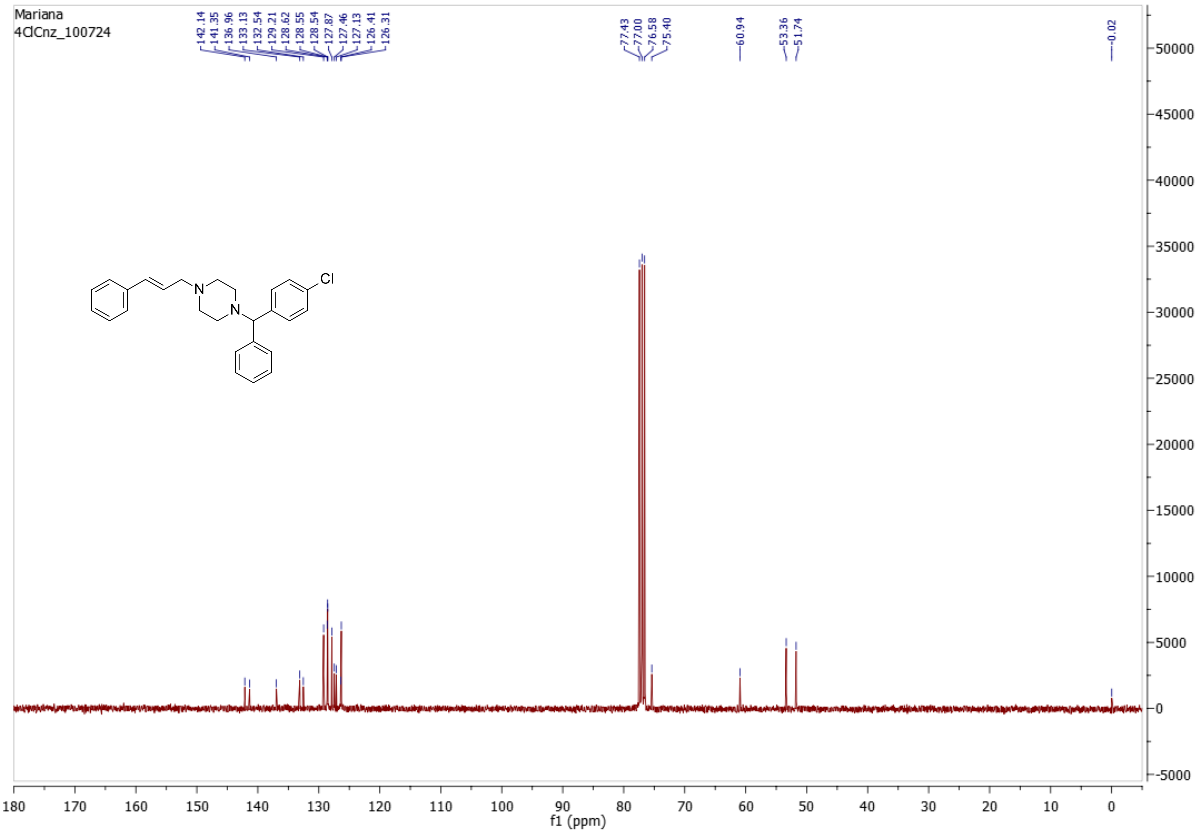

Figure S6. ^{13}C-NMR spectrum of compound **2**

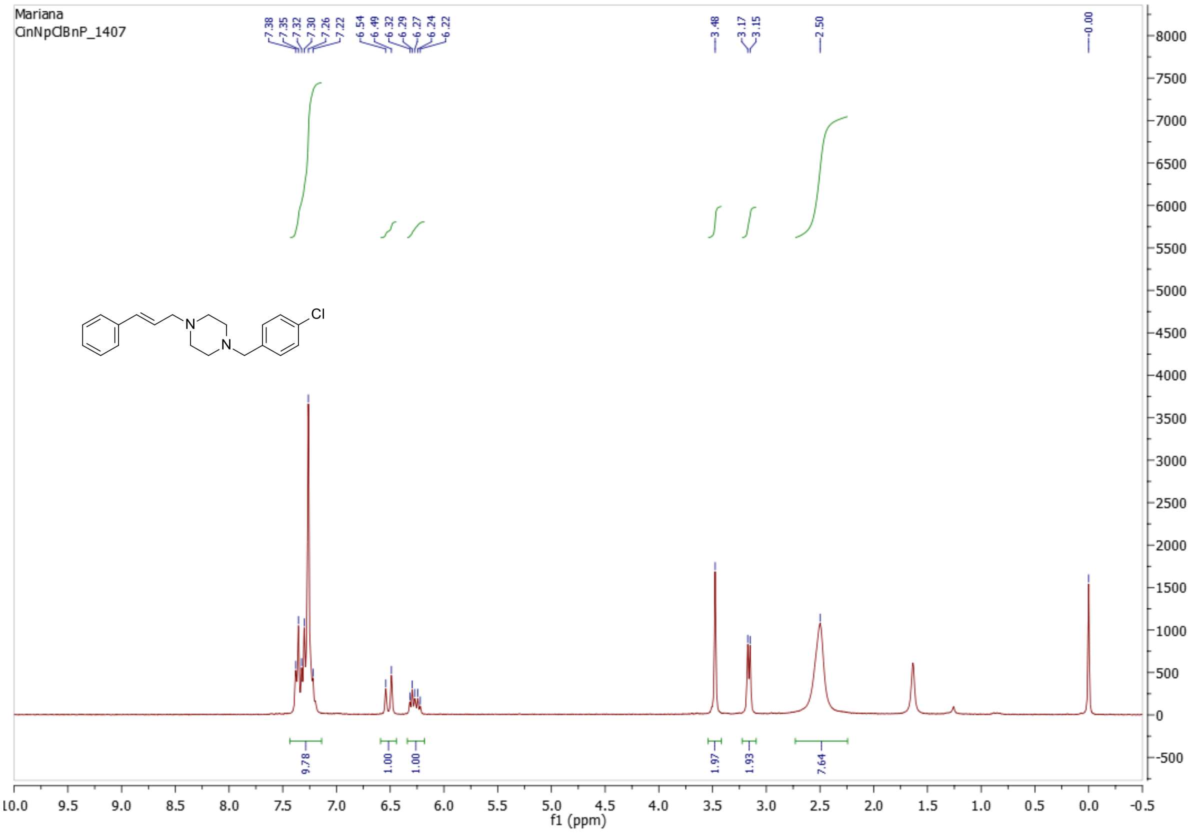

Figure S7. ^{1}H-NMR spectrum of compound **2a**

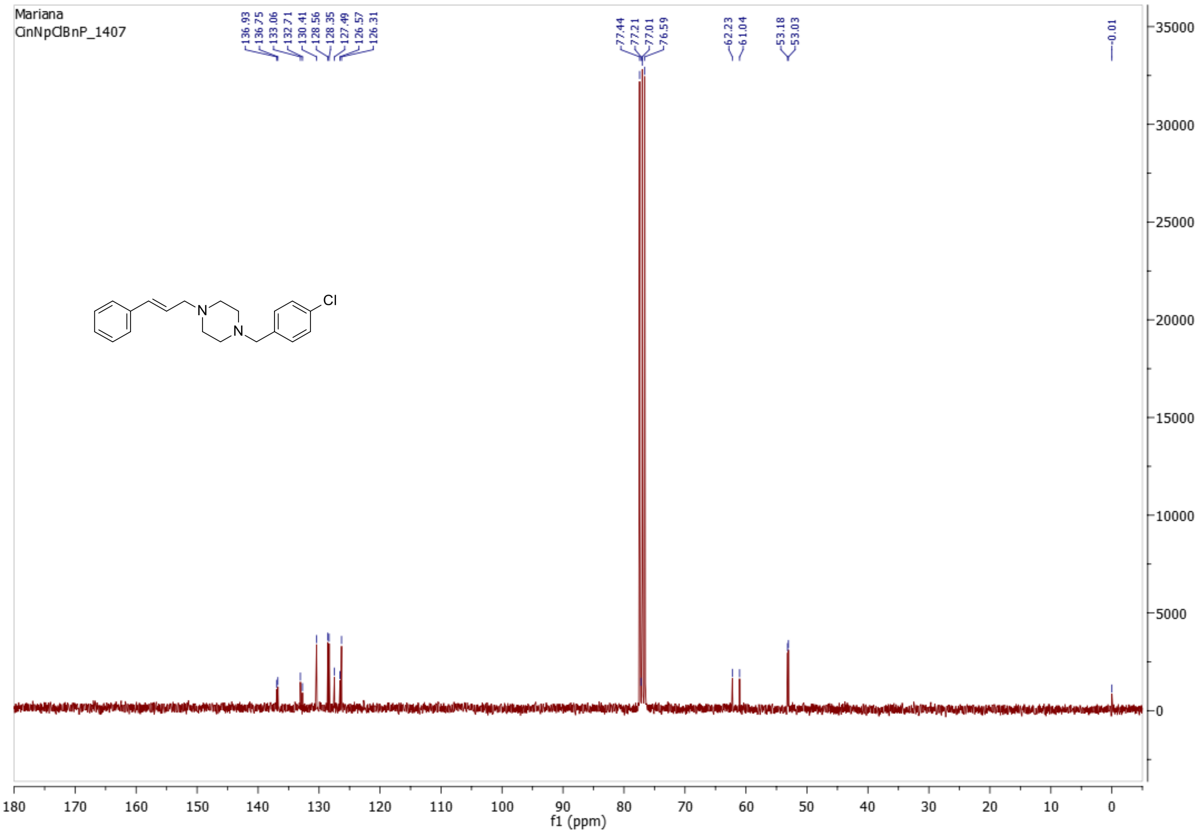

Figure S8. ^{13}C-NMR spectrum of compound **2a**

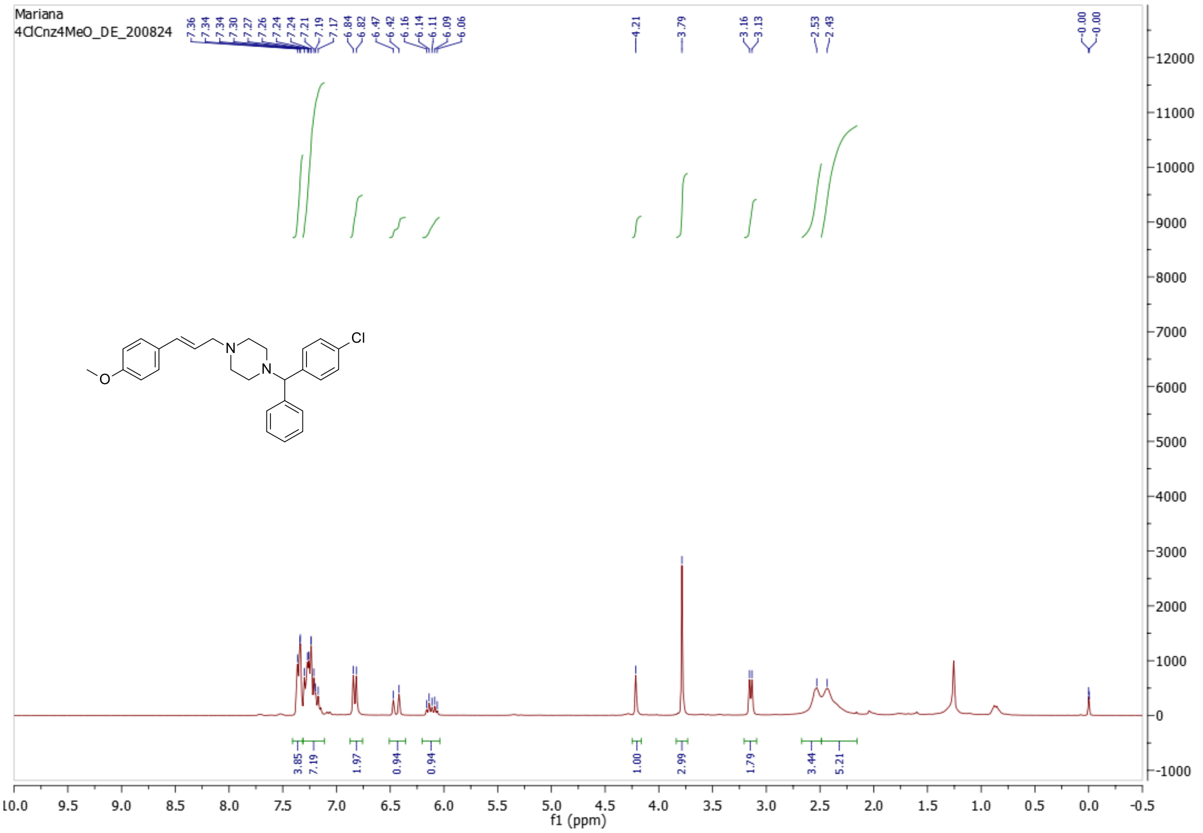

Figure S9. ^{1}H-NMR spectrum of compound **2b**

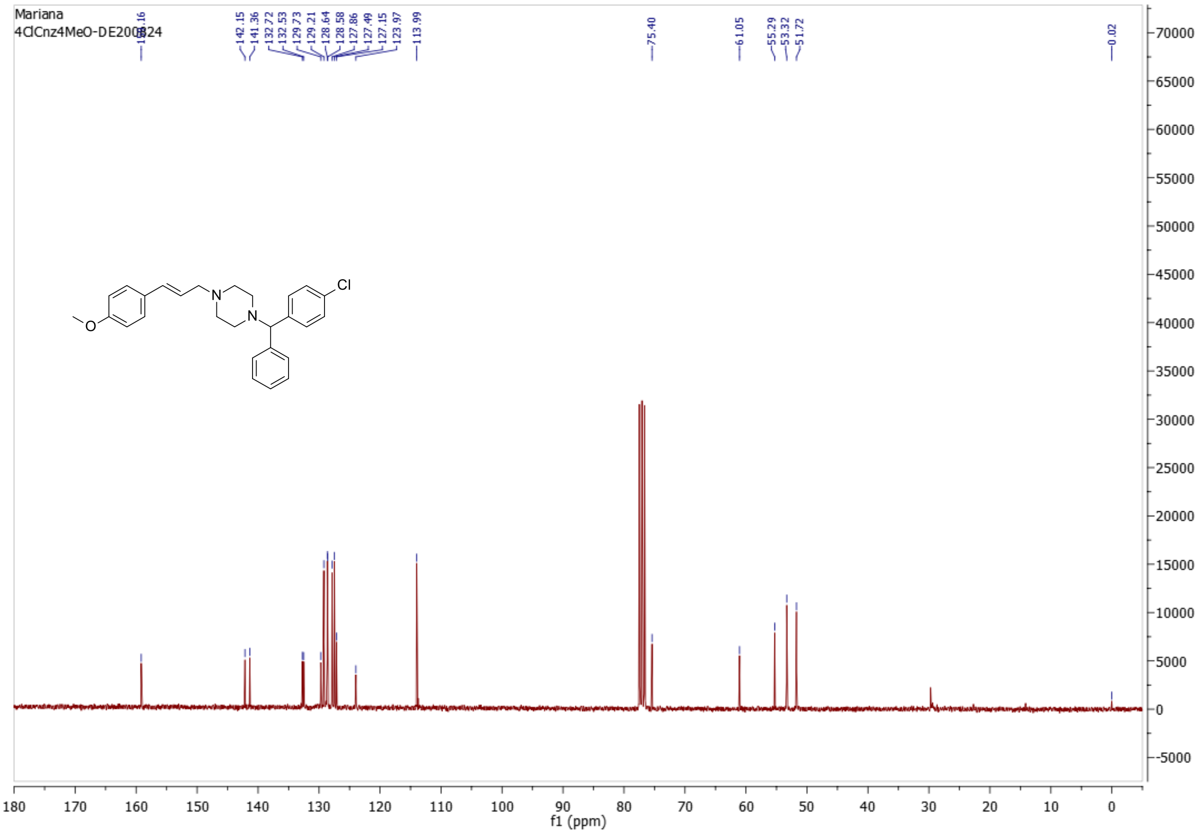

Figure S10. ^{13}C-NMR spectrum of compound **2b**

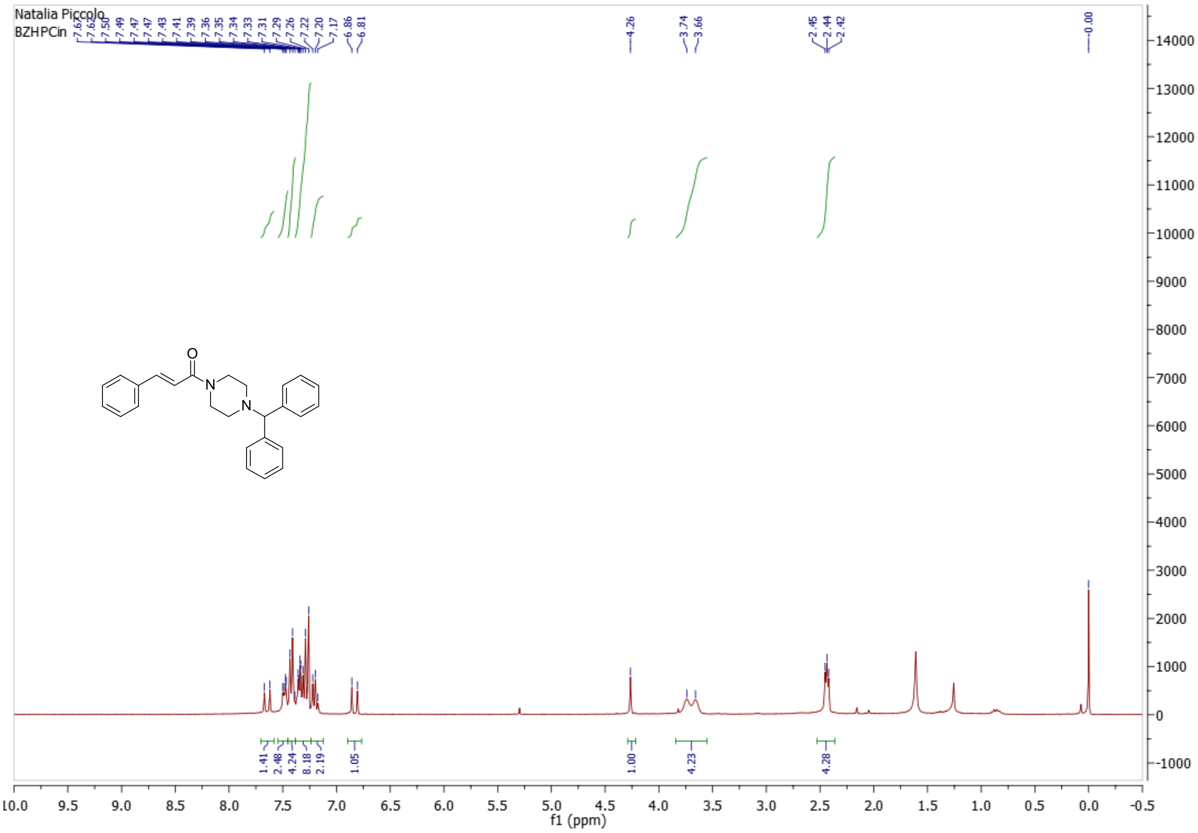

Figure S11. ^{1}H-NMR spectrum of compound **3**

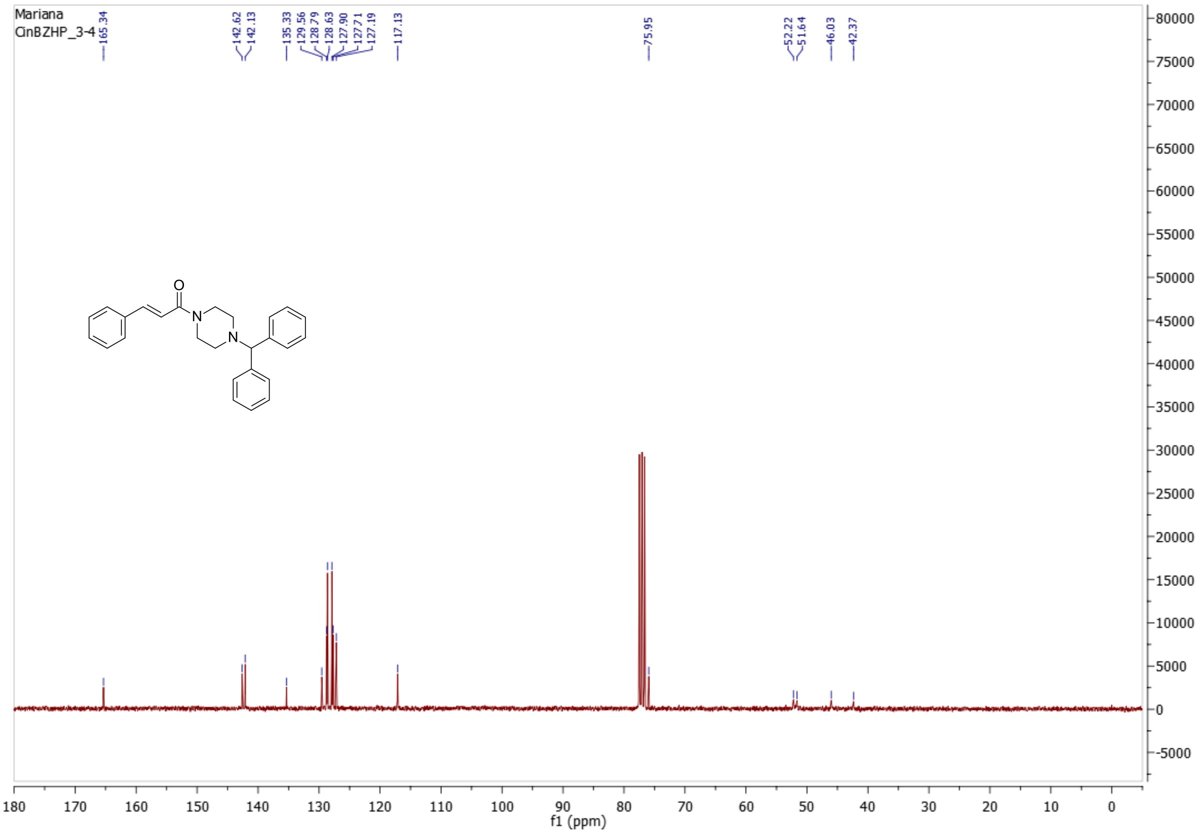

Figure S12. ^{13}C-NMR spectrum of compound **3**

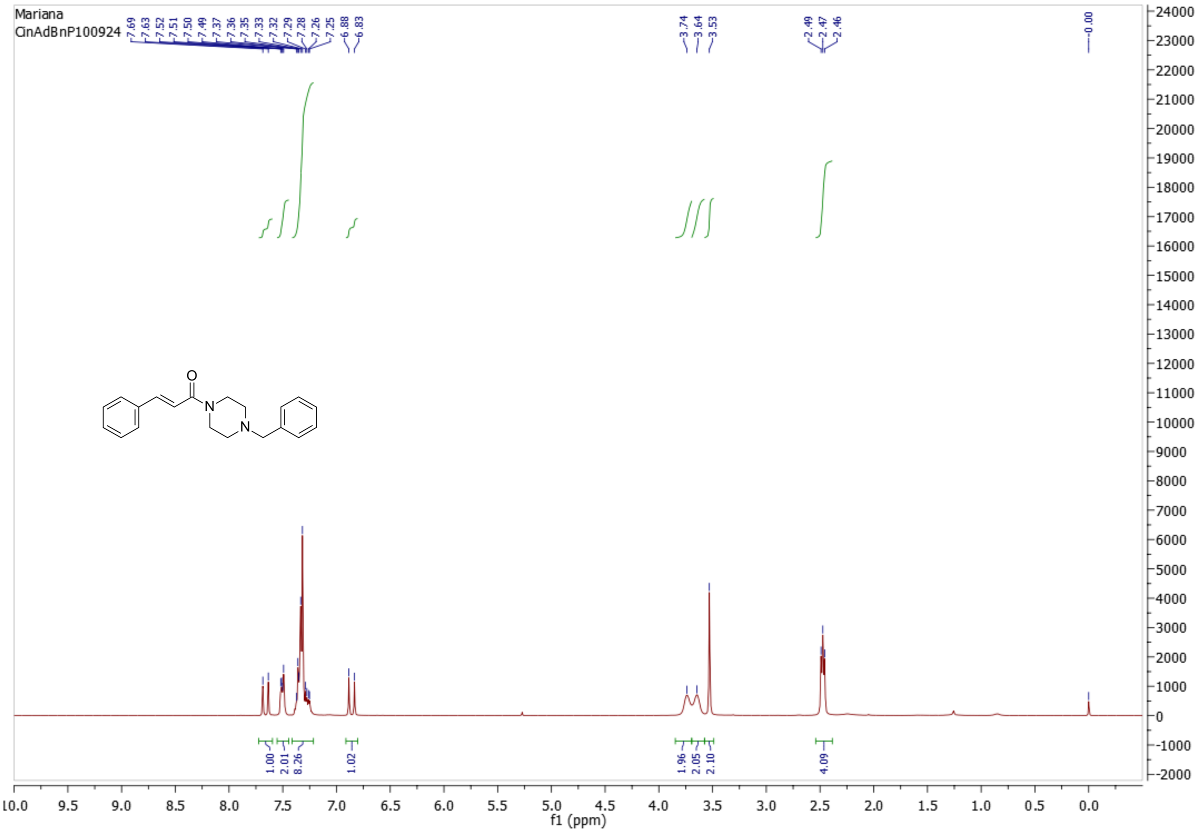

Figure S13. ^{1}H-NMR spectrum of compound **3a**

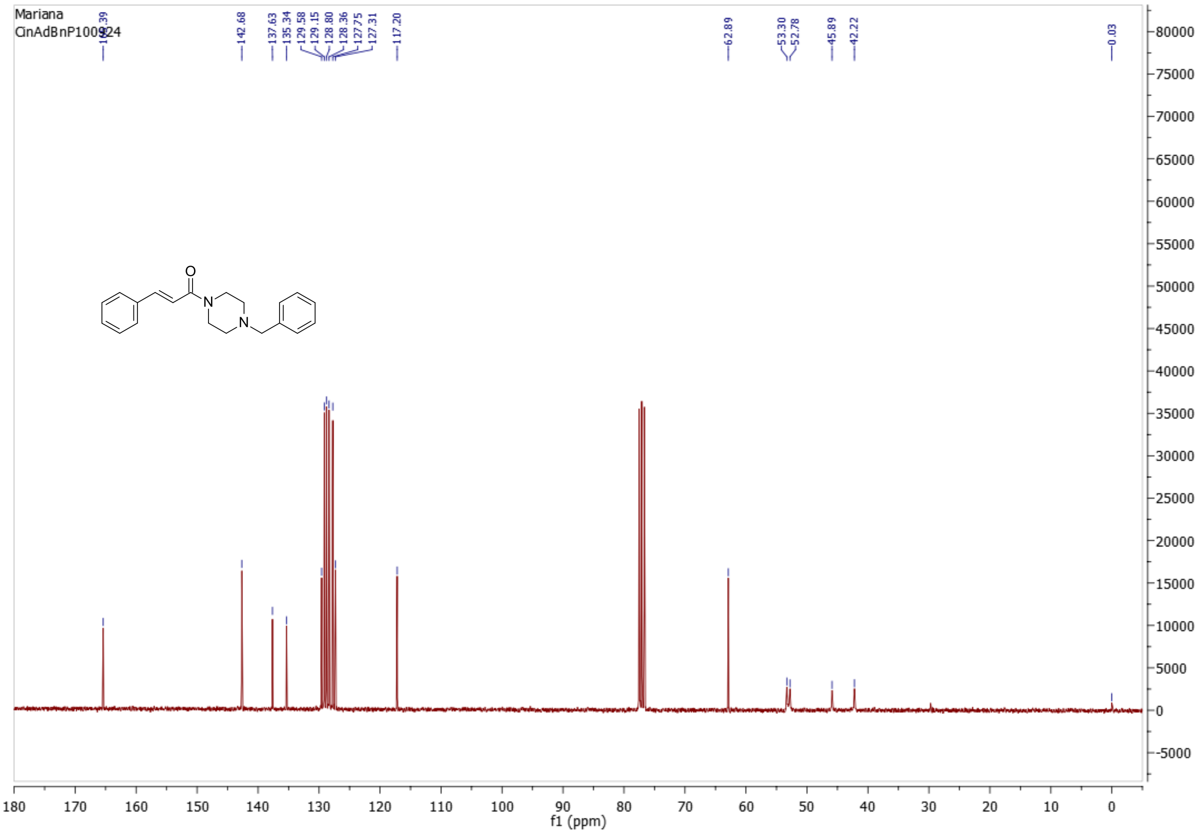

Figure S14. ^{13}C-NMR spectrum of compound **3a**

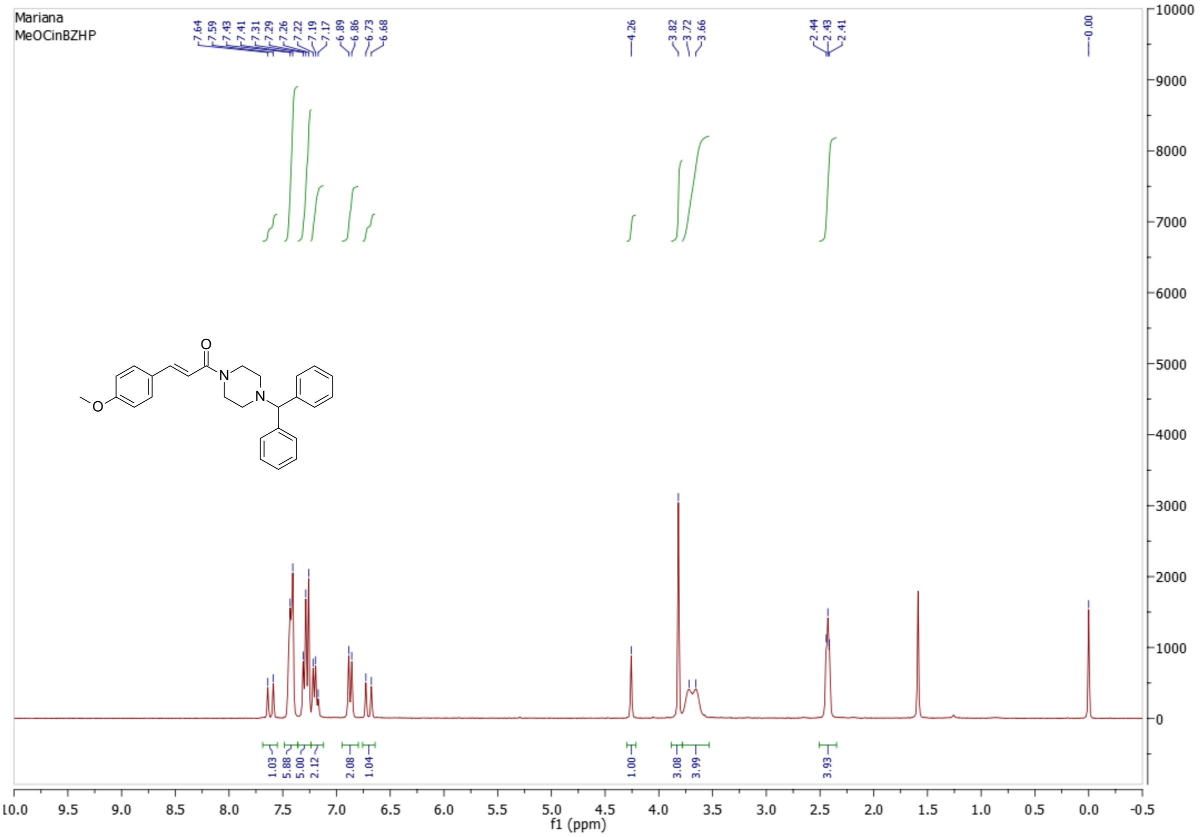

Figure S15. ^{1}H-NMR spectrum of compound **3b**

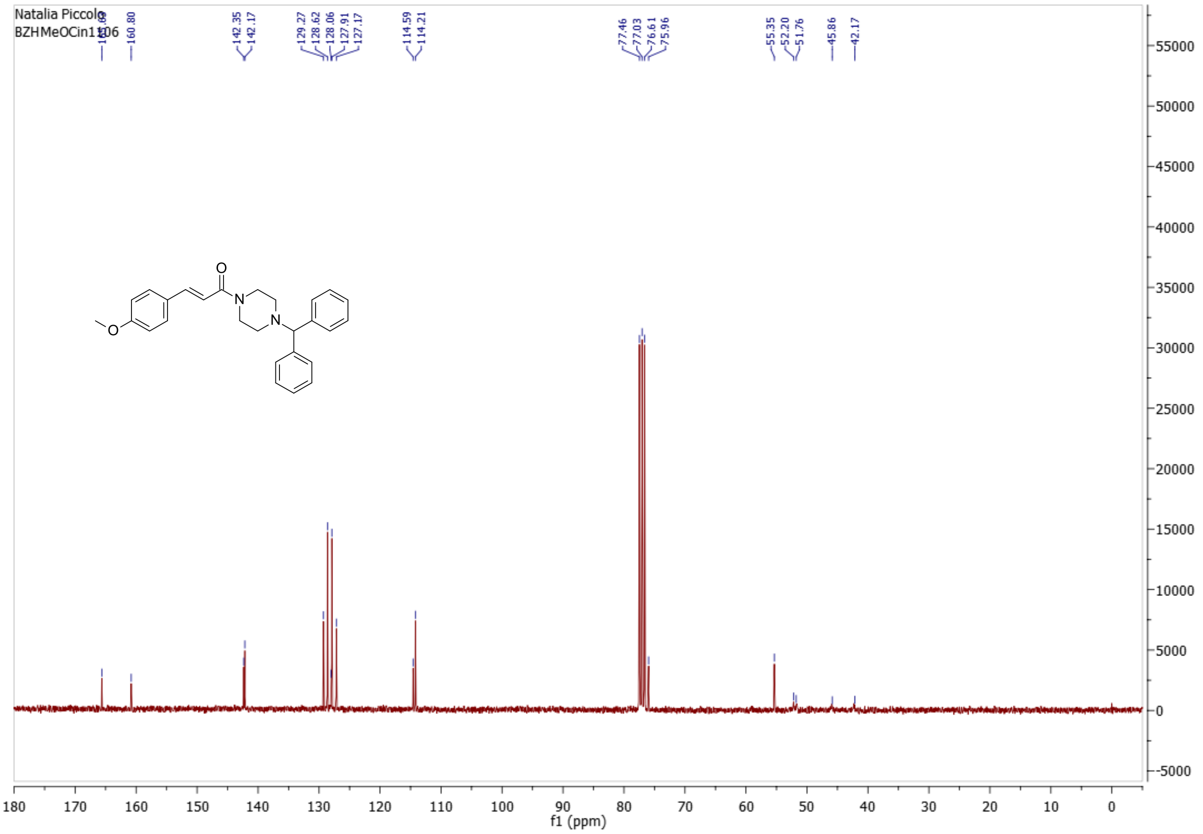

Figure S16. ^{13}C-NMR spectrum of compound **3b**

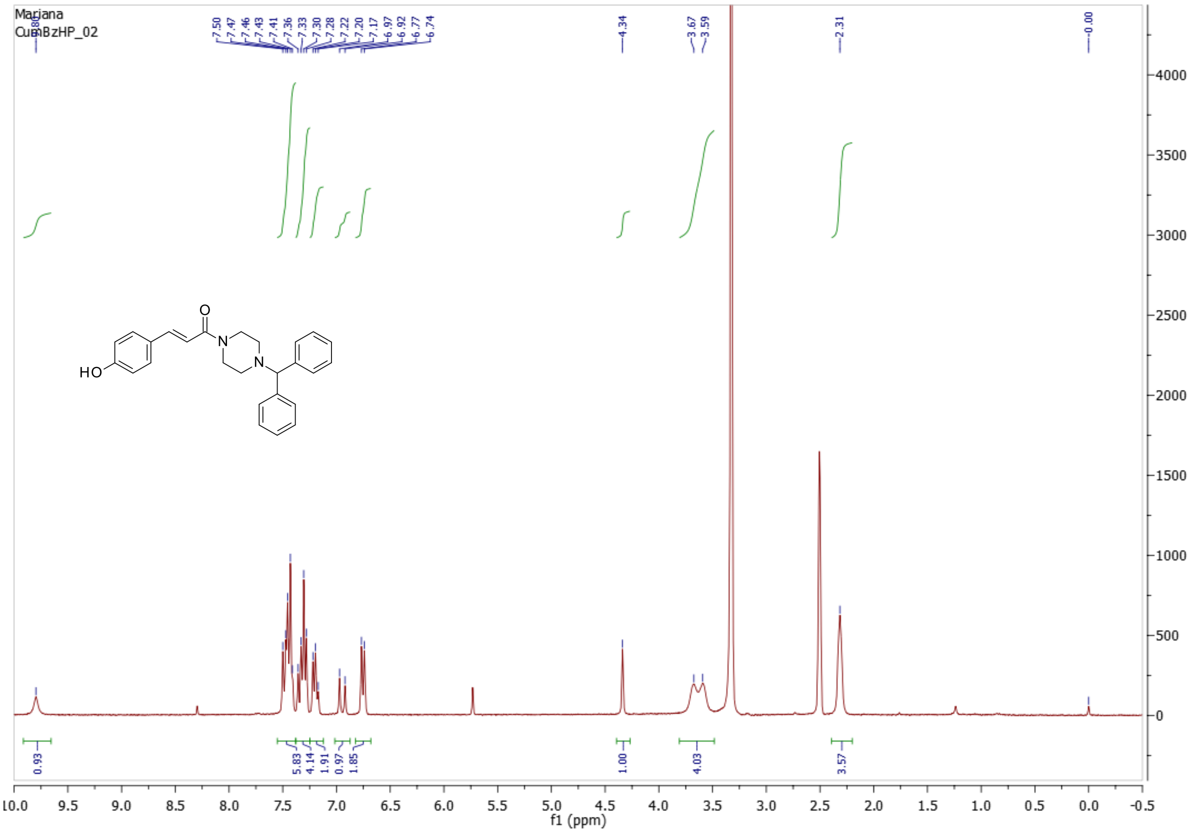

Figure S17. ^{1}H-NMR spectrum of compound **3c**

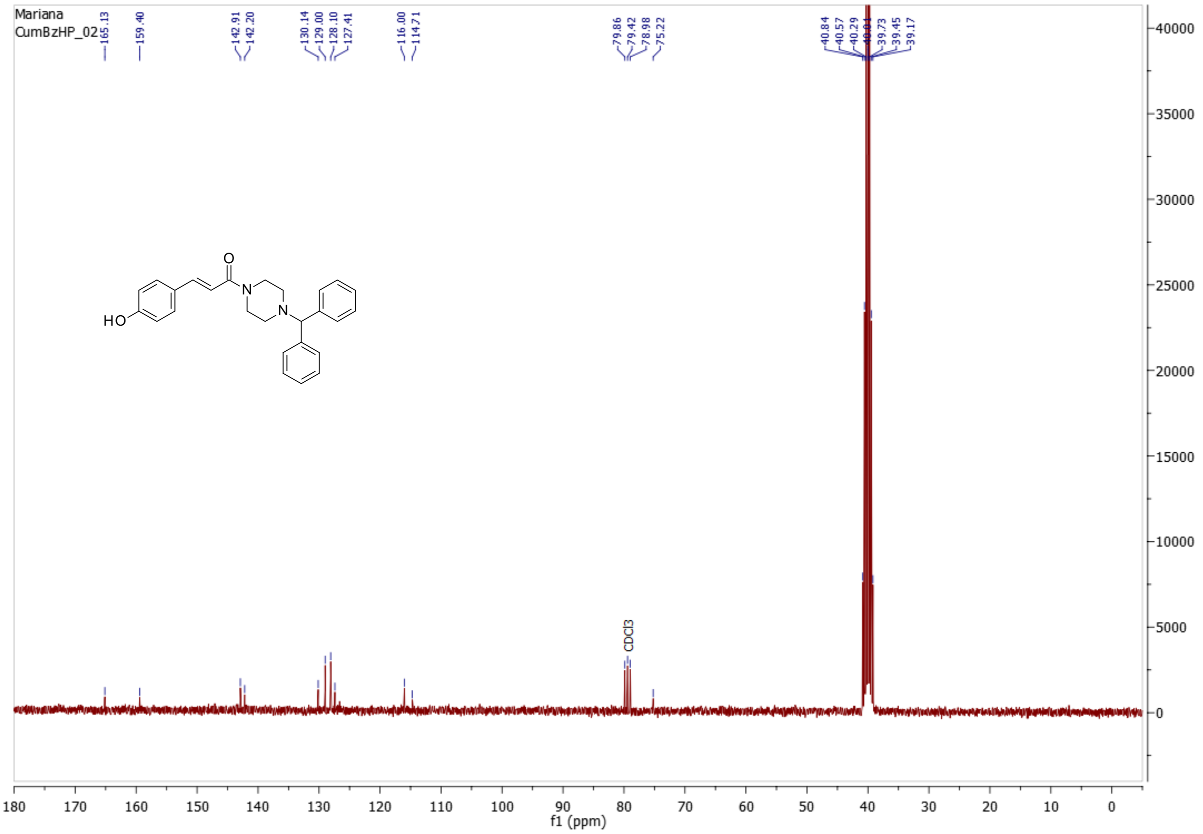

Figure S18. ^{13}C-NMR spectrum of compound **3c**

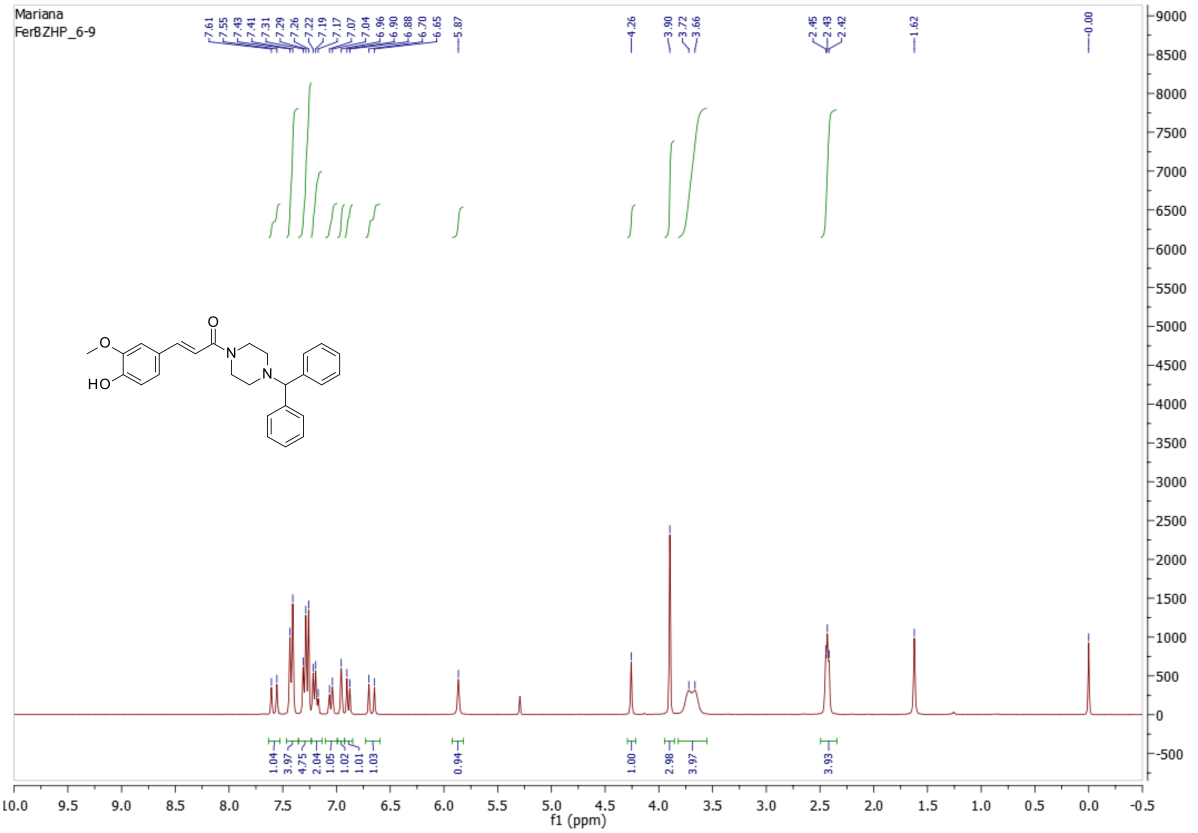

Figure S19. ^{1}H-NMR spectrum of compound **3d**

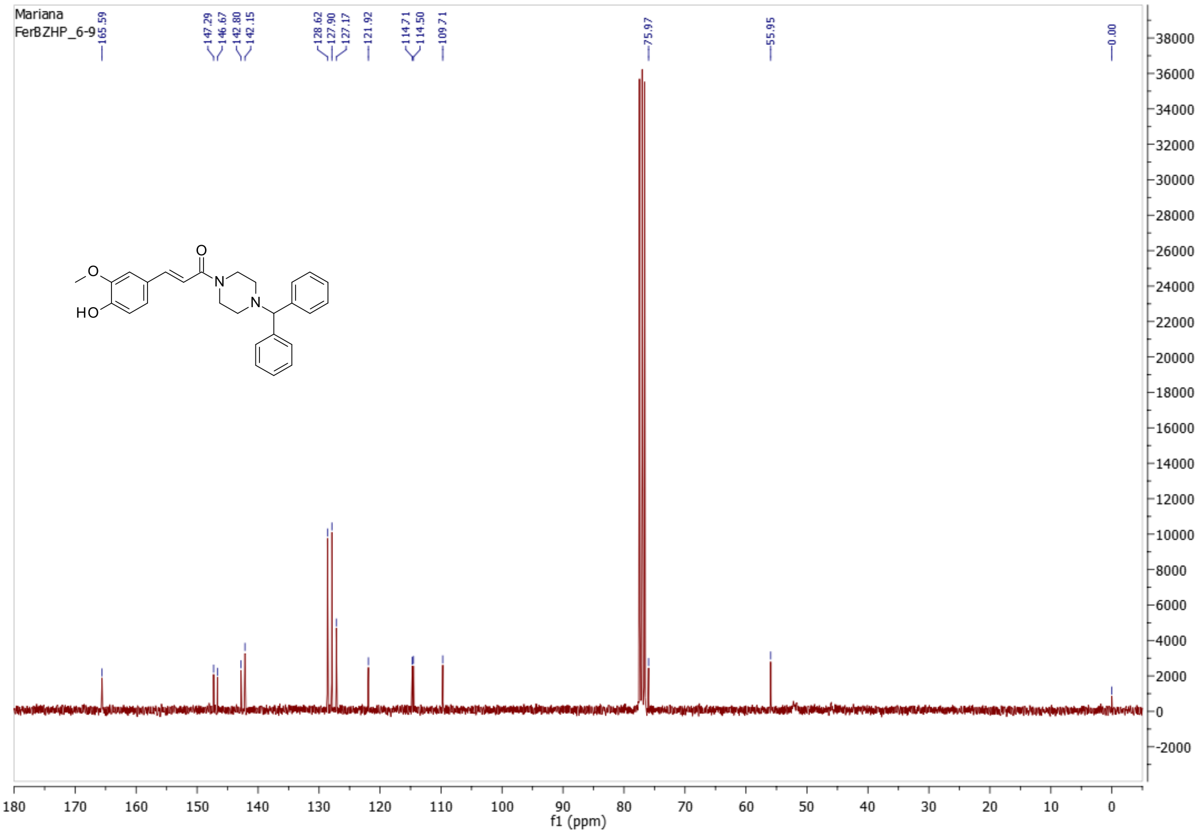

Figure S20. ^{13}C-NMR spectrum of compound **3d**

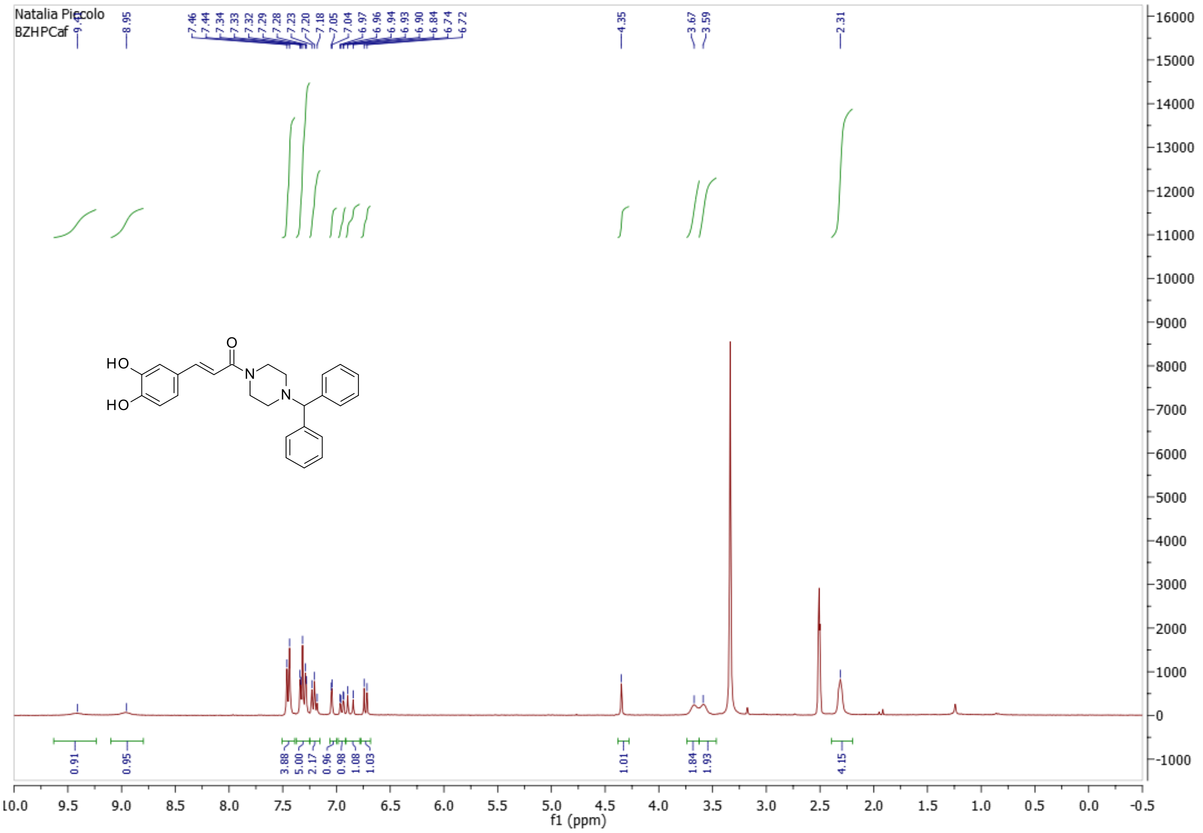

Figure S21. ^{1}H-NMR spectrum of compound **3e**

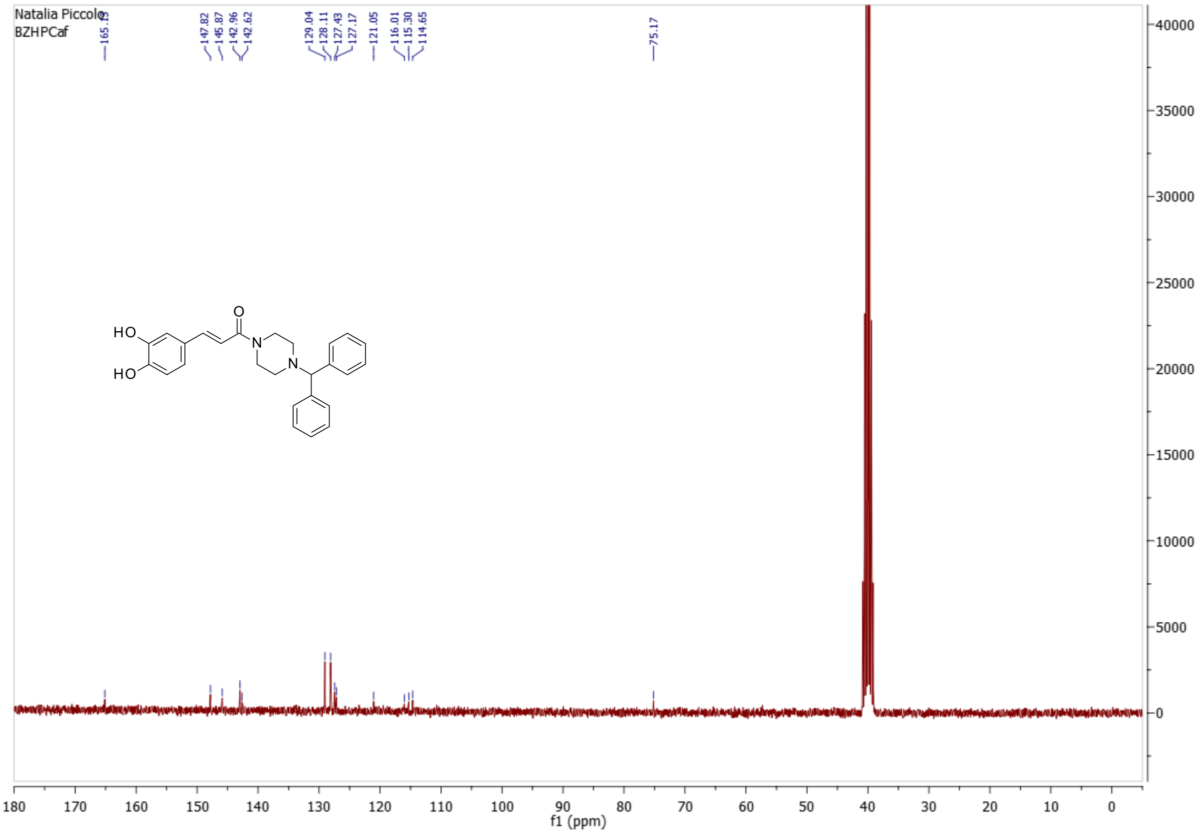

Figure S22. ^{13}C-NMR spectrum of compound **3e**

Table S1. Descriptor's loadings on PC1 and PC2 in the PCA.

Descriptor	PC 1	PC 2
MW	0.4667	0.11439
Fsp3	-0.27353	-0.4469
RB	0.43497	0.0052002
MR	0.47853	0.038669
TPSA	0.15831	0.6346
Log P	0.3064	-0.52263
Log S	-0.4126	0.33137
